# Phenotypic Florfenicol Resistance in *floR*‐Harbouring *Flavobacterium psychrophilum* Isolates (CC‐ST90): A Case Study From Danish Freshwater Rainbow Trout Aquaculture

**DOI:** 10.1111/jfd.70255

**Published:** 2026-07-23

**Authors:** Valentina L. Donati, Lone Madsen

**Affiliations:** ^1^ Section for Fish and Shellfish Diseases National Institute of Aquatic Resources, Technical University of Denmark Kongens Lyngby Denmark

**Keywords:** AMR, antimicrobial resistance, *Flavobacterium psychrophilum*, *flo*R, florfenicol, rainbow trout

## Abstract

Monitoring antimicrobial resistance (AMR) in fish pathogens is essential for supporting sustainable aquaculture. In this study, susceptibility testing and genome sequencing were used to characterize the AMR profile of seven *Flavobacterium psychrophilum* originating from Danish rainbow trout farms (2019–2022). To establish epidemiological context and baseline virulence, isolates were also subjected to genome‐based Multi‐Locus Sequence Typing (MLST), serotyping and, for a subset, in vivo LD_50_ testing. The analysis revealed changes in the susceptibility to antimicrobials used in Danish aquaculture. Most importantly, reduced susceptibility to florfenicol was observed in two isolates originating from a farm experiencing ineffective treatment (disc diffusion diameter of 7–8 mm, MIC > 16 μg mL^−1^). These isolates (serotype Th) belonged to a clonal complex previously unreported in the Nordic countries (CC‐ST90), and the genome analysis revealed a *floR* gene (413 aa) lying in a large genomic island, putatively conferring this property. The high diversity of the *floR* GC content (57.4%) from the *floR*‐harbouring genomes (32.7%–32.8%) suggested its recent acquisition. Since florfenicol is the drug of choice for this infection in Denmark, these observations are highly relevant. However, in vitro lab and in vivo animal experiments are needed to confirm the activity of *floR* and support the clinical relevance of these findings. Also, a larger bacterial panel is needed to evaluate potential trends across farms/regions.

## 1 Introduction

In the aquaculture sector, fish production and consumption have increasingly grown in recent years, and they are predicted to rise in the future, especially when considering their key role as future food systems in the visionary strategy ‘Blue transformation’, focused on the expansion and intensification of sustainable aquaculture (FAO 2022, 2024). Fish pathogens and the diseases they cause as well as the emergence of antimicrobial resistance (AMR) represent critical challenges for the expansion of a sustainable aquaculture sector (FAO 2024). In the EU One Health Action plan against AMR, the creation of surveillance and monitoring programs for AMR together with an improved knowledge on detection and the development of robust and effective preventive and control measures are identified as of crucial importance to fight the spread of AMR and to support a prudent and informed use of antimicrobials (European Commission 2017; European Parliament 2023). At European level, technical specifications for a survey on AMR in bacteria, e.g., *Aeromonas* spp. from external organs of healthy finfish aquaculture animals (not pathogens for aquaculture animals) have been prepared and represent a starting point for harmonized AMR monitoring in aquaculture productions in relation to environmental risk and human consumption. In the technical specification, it is also suggested that monitoring of AMR in pathogens for aquaculture animals would be complementary even if this does not concern the purpose of the mandate (European Food Safety Authority (EFSA) et al. 2024).

The use of antibiotics in Danish aquaculture, both marine and freshwater farms, started in the 1950s in relation to furunculosis outbreaks caused by the bacterial fish pathogen *Aeromonas salmonicida* . At first, oxytetracycline (OXY, 1965‐early 90s) and sulfamerazin (1956–1983) were used for the treatment of bacterial infections in aquaculture. Subsequently, trimethoprim/sulfadiazine (Tribrissen) and oxolinic acid (OXO) have been the licensed drugs for bacterial infections in aquaculture both in marine and freshwater farms since 1984 and 1986, respectively (Dalsgaard 2014).

The first recognized case of infection caused by the Gram‐negative bacterium *Flavobacterium psychrophilum* in Denmark dates to 1985. This bacterium is the etiological agent of Rainbow Trout Fry Syndrome (RTFS) at fry stage and of Bacterial Coldwater Disease (BCWD) from fingerlings to adult (Borg 1948; Dalsgaard and Hørlyck 1990; Lorenzen et al. 1991; Bernardet et al. 1996). Various degrees of disease and mortality in rainbow trout aquaculture have been seen depending on the life stage and size of the fish (Lorenzen and Olesen 1996). Initially, RTFS/BCWD was treated with OXY due to the inefficacy of the two licensed drugs, but its efficacy dropped in the early 90s. Amoxicillin (AMO) was then chosen for a short period (1993–1996) and subsequently florfenicol (FLO) (since 1996 to now) became the drug of choice for the treatment of RTFS (Figure 1). If FLO is not effective in the specific case, OXY and AMO are occasionally used (Bruun et al. 2000; Dalsgaard 2014). Recently, the annual DANMAP2023 report has shown a decreased antimicrobial consumption in marine and freshwater aquaculture for the year 2023 in Denmark (35.6% lower than the average of the previous 5 years) solely due to a reduced use of sulphonamides and trimethoprim. The main classes of antimicrobials used were combination products of sulphonamides and trimethoprim (58.90%), OXO (33.20%) and FLO (7.89%) (Andersen and Sandberg 2023).

**FIGURE 1:**
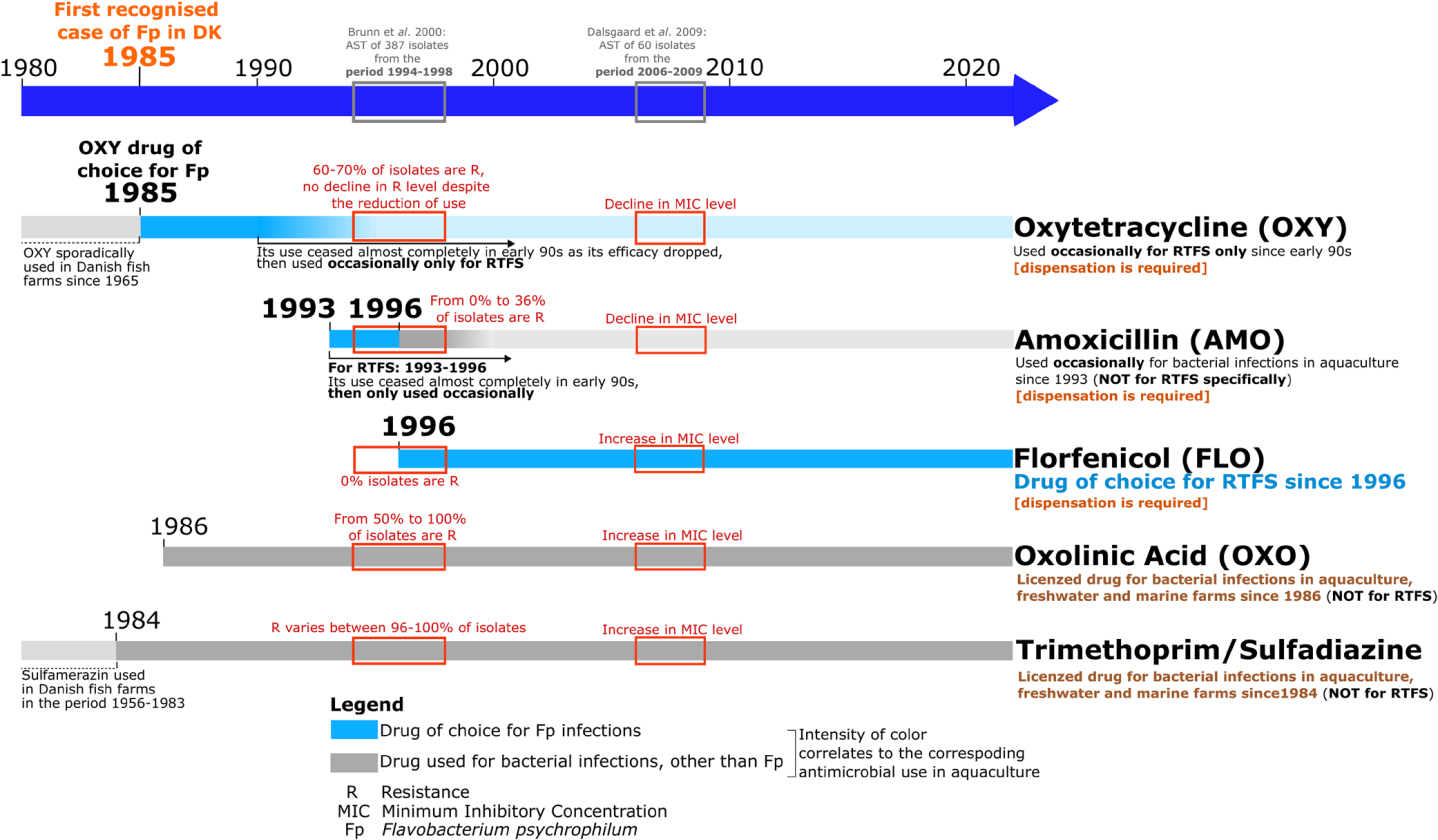
Overview of antimicrobial use in Denmark in relation to RTFS. The first case of *F. psychrophilum* infection in Denmark is dated 1985 (Dalsgaard and Hørlyck 1990). Following a period when oxytetracycline (OXY) and amoxicillin (AMO) were used, florfenicol (FLO) became the drug of choice for this bacterial infection (since 1996). Trimethoprim/Sulfadiazine (Tribrissen) and oxolinic acid (OXO) are also included as they have been the licensed drugs for bacterial infections (not RTFS) in aquaculture both in marine and freshwater farms since 1984 and 1986, respectively. Main results of Antimicrobial Susceptibility Testing (AST) in *F. psychrophilum* isolates in Denmark are highlighted by the red rectangles. Data from: Bruun et al. (2000), Dalsgaard et al. (2009) and Dalsgaard (2014).

In this study, we assess the antimicrobial resistance patterns of seven *F. psychrophilum* isolates from Danish rainbow trout freshwater farms (2019–2022) by combining classical microbiology methods for antimicrobial susceptibility testing (AST) (Bruun et al. 2000; Dalsgaard 2014; CLSI 2020a, 2020b) and whole genome sequencing. Broth micro‐dilution assays (CLSI 2020a, 2020b; with modifications) were included to support disc diffusion AST results as advised when working with aquatic bacteria requiring a prolonged incubation (> 24 h) at a temperature below 22°C such as *F. psychrophilum* (Miranda et al. 2016; Smith et al. 2018).

The panel of *F. psychrophilum* isolates included in this characterization was selected based on isolations connected to research and diagnostic cases. For the latter, we observed within the past 5 years few cases with altered florfenicol susceptibility profiles. Thus, such isolates were selected for further investigations. Serotype characterization, Multi‐Locus Sequence Typing (MLST) and median lethal dose (LD_50_) determination were included in the study to give an epidemiological context and a baseline virulence characterization of the selected panel of bacteria.

## 2 Materials and Methods

### 2.1 Bacterial Isolates

Seven *Flavobacterium psychrophilum* isolates collected from four Danish rainbow trout freshwater farms in the period 2019–2022 were selected (Table 1). These bacteria were isolated in relation to research projects (aim: optimization of infection methods in fish with novel isolates) and diagnostic cases (i.e., with altered florfenicol susceptibility profile) from rainbow trout whose internal organs (spleen, kidney and brain samples) were streaked on tryptone yeast extract salts agar medium (TYES [0.4% tryptone, 0.04% yeast extract, 0.05% CaCl_2_ × 2H_2_O, 0.05% MgSO_4_ × 7H_2_O (pH 7.2) (Holt et al. 1993)] with 1.1% agar referred as TYES‐A). After 4–5 days at 15°C, single colonies of *F. psychrophilum* were identified, confirmed by Matrix‐Assisted Laser Desorption/Ionization Time Of Flight (MALDI‐TOF) Mass Spectrometry (MS) (Jansson et al. 2020) and sub‐cultivated in TYES broth medium (referred as TYES‐B; incubation at 15°C and 115 RPM for 48 h) for −80°C bacterial stocks preparation (TYES‐B and glycerol (15%–20%)) and further confirmation of cultures' purity (plating on TYES‐A and blood‐agar (BA) and incubation at 15°C and 20°C, respectively) (Dalsgaard and Madsen 2000).

**TABLE 1:** *F. psychrophilum* isolates included in the study and overview of analysis performed.

Isolate	Country	Farm	Year	Fish host	Fish size	Outbreak case	Tissue	AST	Serotype	WGS	LD_50_ exp.	Source or publication
**22‐344‐2B**	Denmark	A	2022	*O. mykiss*	3–8 g	Yes	Kidney	x	x	x	N/A^a^	This study
**22‐344‐3B**	Denmark	A	2022	*O. mykiss*	3–8 g	Yes	Kidney	x	x	x	N/A^a^	This study
**21‐9705‐2**	Denmark	A	2021	*O. mykiss*	2–3 g	Yes^c^	Kidney	x	x	x	x	This study
**20‐8139‐2B**	Denmark	A	2020	*O. mykiss*	5 g	Yes^c^	Spleen	x	x	x	x	This study
**21‐9697‐3A1**	Denmark	B	2021	*O. mykiss*	0.2 g, 2.9 cm	Yes	Kidney	x	x	x	x	This study
**20‐1298‐14A G**	Denmark	C	2020	*O. mykiss*	17–30 cm	No^d^	Kidney	x	x	x	x	This study
**19‐12880‐80A**	Denmark	D	2019	*O. mykiss*	N/A	No^e^	Kidney	x	x	x	x	This study
160401‐1/5N	Denmark	E	2016	*O. mykiss*	< 1 g	Yes	Kidney	x	x	x^b^	x	Sundell et al. (2019)
950106‐1/1	Denmark	F	1995	*O. mykiss*	0.4 g	Yes	Spleen	x	x	x^b^	x	Madsen and Dalsgaard (1999)
NCIMB1947	USA	—	1947	*O. kisutch*	N/A	N/A	Kidney	x	x	x^b^	N/A	Bernardet et al. (1996), Bernardet and Bowman (2006)

*Note:* The underlined isolates were collected from the same diagnostic case but from two different fish. The well‐characterized *F. psychrophilum* isolates 950106‐1/1, 160401‐1/5N and NCIMB1947 were included as references. Farm A, B and C are recirculated freshwater aquaculture facility (RAS). For fish size, weight and/or length of fish are indicated.

Abbreviations: AST, antimicrobial susceptibility testing; N/A, not applicable (not performed); WGS, whole genome sequencing; x, performed.

^a^ The bacteria were isolated after the LD_50_ experiments had been done.

^b^ Previously sequenced in other studies, the genomes were downloaded from NCBI (see Table S2).

^c^ Samples were received after 10–16 days of ineffective florfenicol treatment in connection with a disease outbreak on the farm.

^d^ The isolate 20‐1298‐14A G was isolated from a fish without clinical signs in a fish‐batch infected with *Yersinia ruckeri* . Fp and Yr were also found in other fish sampled from the same batch. G stands for gliding isolate.

^e^ It originates from naïve fish in cohabitation with fish transported from a fish farm to our facilities in relation to other projects, which then developed skin lesions and thus it was sampled.

As references, the two Danish isolates *F. psychrophilum* 950106‐1/1 (serotype Fd, virulent, isolated in 1995) and *F. psychrophilum* 160401‐1/5N (serotype Th, virulent, isolated in 2016) were included in the study together with the American strain NCIMB1947 (Madsen and Dalsgaard 1999, 2000; Dalsgaard and Madsen 2000; Bernardet and Bowman 2006; Sundell et al. 2019). *Escherichia coli* ATCC25922 and *Aeromonas salmonicida* subsp. *salmonicida* ATCC33658 were included in the broth micro‐dilution antimicrobial test as quality controls according to Clinical & Laboratory Standards Institute (CLSI) guidelines (CLSI 2020a, 2020b).

### 2.2 Antimicrobial Susceptibility Testing (AST)

#### 2.2.1 Selection of Antimicrobial Agents and Terminology

AST was performed following the guidelines proposed by the CLSI (CLSI guidelines VET03), at first by disc diffusion assays (Bruun et al. 2000; Dalsgaard 2014; CLSI 2020a). Broth micro‐dilution method was further included to support the findings, but a modification to the CLSI suggested protocol was in this case needed to support bacterial growth (CLSI 2020a, 2020b) (Section 2.2.3). Antimicrobial agents used in Danish aquaculture were selected: florfenicol, oxolinic acid, potentiated sulphonamides, amoxicillin and oxytetracycline (Bruun et al. 2000; Dalsgaard 2014). In the case of broth micro‐dilution AST, the custom‐made dry panels were kindly provided by Cefas (Weymouth, UK) and contained a larger set of antimicrobials, previously tested in other fish pathogens (Smith et al. 2023). EUCAST abbreviations codes for antimicrobials were used (https://www.eucast.org/news‐detail/eucast‐system‐for‐abbreviating‐antimicrobial‐agents‐update/).

For data interpretation of disc diffusion AST, we relied on the routinely used interpretive criteria of characterizing *F. psychrophilum* isolates as susceptible, intermediate or resistant (Dalsgaard 2014) as no interpretive categories and zone diameter ECVs (epidemiological cut‐off values) are officially available (CLSI 2020b). For data interpretation of broth micro‐dilution assays, interpretive categories and MIC (minimal inhibitory concentration) ECVs are available for florfenicol, oxytetracycline, oxolinic acid and enrofloxacin. Thus, isolates were characterized as wild‐type (WT, isolates with no acquired resistance detectable in the phenotype nor reduced susceptibility to the selected antimicrobial) or non‐wild‐type (NWT, isolates with acquired resistance or reduced susceptibility to the selected antimicrobial) for these four antimicrobial agents (CLSI 2020b). Quality control strains were included in the broth micro‐dilution AST to support the validity of the test as well as the utilization of the established ECVs despite the medium modification for inoculum preparation. No clinical breakpoints are available for this species (CLSI 2020a, 2020b).

#### 2.2.2 Disc Diffusion AST

Disc diffusion AST were performed as follows (CLSI guidelines VET03) (Bruun et al. 2000; Dalsgaard 2014; CLSI 2020a). A starter broth culture (5 mL of TYES‐B) was prepared by the inoculation of material from the −80°C stocks and incubated for 48 h before plating on TYES‐A (15°C, 115 RPM). After 5 days, 3–5 colonies were picked and transferred into 5 mL TYES‐B and incubated at 15°C (115 RPM) for 72 h. The 72‐h broth culture was then used as inoculum (approximately 1 × 10^8^ CFU (Colony Forming Units) mL^−1^, 0.5 McFarland) and a cotton swab was used to streak the bacteria on 1:7 diluted Muller‐Hinton agar (DMHA) plus 5% foetal bovine serum (FBS). Plates were read after 5 and 7 days (growth dependent) of incubation at 15.9°C ± 0.1°C. Disc diffusion tests were performed at least twice for each isolate. As Quality Controls, the two well‐characterized Danish isolates *F. psychrophilum* 950106‐1/1 and 160401‐1/5N were included together with the American strain NCIMB1947. No other officially recognized Quality Control isolate exists for this test (CLSI 2020a). The antimicrobials used for the test [Thermo Scientific Oxoid Antimicrobial Susceptibility Test (AST) discs (6 mm) (Landsmeer, the Netherlands)] were: Florfenicol (FLO, 30 μg, catalogue no. CT1754B), Oxolinic acid (OXO, 2 μg, catalogue no. CT0181B), Trimethoprim/Sulphamethoxazole 1:19 (TRS, 25 μg, catalogue no. CT0052B), Amoxicillin (AMO, 25 μg, catalogue no. CT0061B), Oxytetracycline (OXY, 30 μg, catalogue no. CT0041B). Indication of resistance was evaluated according to Dalsgaard (2014): resistance to OXO when diameter ≤ 30 mm; resistance to TRS when diameter ≤ 30 mm; resistance to OXY when diameter ≤ 42 mm; resistance to FLO when diameter ≤ 30 mm; resistance to AMO when diameter ≤ 31 mm.

#### 2.2.3 Broth Micro‐Dilution AST

To quantitatively measure the in vitro activity of various antimicrobial agents, we performed a broth micro‐dilution AST for MIC determinations following CLSI guidelines VET03 (testing at 18°C ± 2°C for 92–96 h) with one modification: sterile TYES‐B for inoculum dilutions was used instead of the recommended diluted cation‐adjusted Mueller‐Hinton Broth (CAMHB) (CLSI 2020a, 2020b). This modification was made because diluted CAMHB (4 g L^−1^, pH 7.3 at room temperature) did not support the growth of the selected panel of *F. psychrophilum* isolates in our tests (Mueller Hinton Broth 2, NutriSelect Plus, Cat no. 90922, Millipore, Merck, Germany). A starter broth culture (5 mL of TYES‐B) was prepared by the inoculation of material from the −80°C stocks and incubated for 72 h before plating on TYES‐A (15°C, 115 RPM). After 4 days, 4–5 colonies with the same morphology were picked for each isolate and re‐suspended in sterile TYES‐B for inoculum preparation (turbidity equivalent to 0.5 McFarland, approximately 1 × 10^8^ CFU mL^−1^). A 96 h‐old starter broth culture was utilized for the inoculum preparation for four isolates (22‐344‐2B, 22‐344‐3B, 21‐9705‐2). *Escherichia coli* ATCC25922 and *Aeromonas salmonicida* subsp. *salmonicida* ATCC33658 were included in the test as quality controls (QC) as well as the well‐characterized *F. psychrophilum* 950106‐1/1, 160401‐1/5N and NCIMB1947. Custom‐made 96‐well broth micro‐dilution dried panels (Sensititre AQGNECV, Thermo Scientific, United Kingdom (Smith et al. 2023)) were inoculated with 100 μL of the adjusted inoculum suspension (approximately 5 × 10^5^ CFU mL^−1^) and incubated at 16.4°C ± 0.5°C for 96 h and then MIC values were determined as the lowest antimicrobial concentration where the isolate did not grow. Inoculum suspensions were checked according to CLSI guidelines (i.e., colony counts to ensure a bacterial concentration close to 5 × 10^5^ CFU mL^−1^). The custom‐made plates contained the following antimicrobials: Ampicillin (AMP, 0.015–16 μg mL^−1^), Ceftazidime (CTZ, 0.03–8 μg mL^−1^), Enrofloxacin (ENR, 0.0005–0.25 μg mL^−1^), Florfenicol (FLO, 0.03–16 μg mL^−1^), Gentamicin (GEN, 0.06–8 μg mL^−1^), Meropenem (MER, 0.008–1 μg mL^−1^), Oxolinic acid (OXO, 0.002–1 μg mL^−1^), Oxytetracycline (OXY, 0.015–8 μg mL^−1^), Sulfamethoxazole (SME, 1–512 μg mL^−1^), Trimethoprim/Sulfamethoxazole (TRS, 0.008/0.15–1/19 μg mL^−1^). All plates contained both negative and positive growth controls. A preliminary test including quality controls strains as well as the well characterized *F. psychrophilum* 950106‐1/1 was first performed to test the method in our lab. Consistent results were obtained. According to CLSI guidelines (CLSI 2020a, 2020b), acceptable QC ranges for both QC bacteria are provided for AMP, FLO, OXO, OXY and TRS (1/19) except ENR where QC ranges are provided only for *A. salmonicida* subsp. *salmonicida* ATCC33658 (testing at 18°C ± 2°C for 92–96 h).

### 2.3 Median Lethal Dose (LD_50_ ) Assessed by in vivo Experimental Infections

#### 2.3.1 Fish, Experimental Set Up and Procedures

Rainbow trout ( *Oncorhynchus mykiss* ) eyed eggs were purchased at a Danish commercial fish farm negative for Infectious Pancreatic Necrosis (IPN), Viral Hemorrhagic Septicemia (VHS), Infectious Haematopoietic Necrosis (IHN) and Bacterial Kidney Disease (BKD). Eyed eggs were iodophore disinfected (10 min treatment; 100 ppm active iodine; 1% Actomar K30 (Desag AF, Uster, Switzerland)), hatched, and fish grown at the BLUE Unit facilities of the Section for Fish and Shellfish Diseases (DTU Aqua, Kgs. Lyngby, Denmark) (https://www.aqua.dtu.dk/english/facilities/fish‐shellfish‐diseases). Initially raised in an *ad hoc* area to produce experimental pathogen‐free rainbow trout with its own recirculation system, fish were transferred to a high‐containment laboratory area used for experimental challenges (flow‐through system) and divided randomly in 8‐L tanks, each with its own air‐supply and inlet/outlet for water (water temperature constant at 12°C). The fish were fed twice a day (morning and afternoon) at 2% of their weight per day. Aquaria were cleaned and the water exchanged daily.

Five of the new isolates were tested in animal experiments for LD_50_ determinations: two isolates with resistance/reduced susceptibility to florfenicol originating from farm A and associated with ineffective florfenicol treatment in the field (21‐9705‐2 and 20‐8139‐2B), and three other isolates originating from either farm B, C or D. The Danish reference strains, *F. psychrophilum* 950106‐1/1 and 160401‐1/5N, were included as quality controls. Based on previous research (Sundell et al. 2019), an in vivo experimental infection was designed to characterize the median lethal dose (LD_50_) of the selected isolates in rainbow trout fry (588 fish of 1–2 g). For each of the seven isolates, two aquaria were set up (duplicates, 14 aquaria in total) and, in each aquarium, fish infected with six different doses with a separation factor of 10 (from 10^7^ to 10^2^ CFU fish^−1^) of the same isolate were placed together with negative control fish (sterile TYES‐B) (6 doses + negative control, 6 fish/dose, 42 fish/aquarium, 2 aquaria/isolate). For each bacterial dose, a specific type of fin clipping (pectoral fin, pelvic fins, anal fin, adipose fin, dorsal or ventral tail fin) was performed to allow fish recognition during the trial. Negative control fish were not fin clipped.

For bacterial preparations, *F. psychrophilum* was grown as described by Madsen and Dalsgaard (1999). Prior to infection procedures, a 44‐46 h old culture (~10^9^ CFU mL^−1^) was diluted by serial tenfold dilutions with sterile TYES‐B and injected by intraperitoneal (IP) injection (50 μL; 10^7^–10^2^ CFU fish^−1^). Sterile TYES‐B (50 μL) was injected into negative control fish. IP was selected for the LD_50_ experiments due to its reproducibility in relations to experimental *F. psychrophilum* infections in rainbow trout (Madsen and Dalsgaard 1999). Finally, appropriate dilutions of the 44–46 h old culture were also performed for CFU enumeration before and after infection procedures.

Prior to the infection procedure, fish were not fed for 24 h. On infection day, fish were anaesthetised by immersion (3‐aminobenzoic acid ethyl ester, MS‐222), fin clipped and then challenged by IP. Several times per day after the IP injection challenge, aquaria were inspected and dead and moribund fish were collected from each aquarium. Moribund fish and fish that were alive at the end of the experiment were euthanized by an overdose of MS‐222 in water. Date, aquaria number and type of fin clipping were recorded. Dead and euthanized fish were weighed, and their length measured, and bacteriological examination of each fish was performed. The experiments were terminated 21 days post infection.

To perform the bacteriological examination of dead/euthanized fish, samples from fish organs (spleen, kidney, brain) were streaked on TYES‐A and incubated at 15°C for 5 days. TYES‐A plates were read to confirm the presence of *F. psychrophilum* in infected fish (presence of yellow colonies, randomly checked with MALDI‐TOF MS). TYES‐A plates were re‐inspected after 3–4 weeks of incubation at 15°C.

#### 2.3.2 Statistical Analysis

GraphPad Prism version 10.1.1 for Windows (GraphPad Software, San Diego, CA, United States, www.graphpad.com) was used for performing Kaplan–Meier survival analysis. Data from singular replicate aquaria were merged. For each isolate, LD_50_ was estimated with the Reed‐Muench method (Reed and Muench 1938). The method requires observation of cumulative mortality above and below 50% among the infected groups.

#### 2.3.3 Ethical Statement

The animal study was reviewed and approved by the Animal Experiments Inspectorate of Denmark (Dyreforsøgstilsynet, permission no. 2019‐15‐0201‐00159).

### 2.4 Serotype

Bacterial DNA was extracted as for genome sequencing (see genome sequencing section below) and used as template DNA for the multiplex PCR‐based serotyping scheme developed by Rochat et al. (2017). The 50 μL PCR reaction mixture consisted of 25 μL of the DreamTaq Green PCR Master Mix (2×) (catalogue no. K1081, ThermoFisher Scientific, Denmark), 2 μL of DNA template, the four sets of primers developed by Rochat et al. (2017) (final concentration of 0.3 μM) and RNase free water. The PCR reactions were heated for initial denaturation at 95°C for 10 min, followed by 30 cycles of 95°C for 30 s, 52°C for 30 s, 72°C for 60 s and a final extension at 72°C for 10 min. The PCR products were analysed using Invitrogen E‐Gel Double Comb Agarose Gels with SYBR Safe DNA Gel Stain, 2% (catalogue no.: Invitrogen A42348, Fisher Scientific, Denmark) [15 min] and visualized with the instrument Bio‐Rad GelDoc Go Imaging System (Bio‐Rad GelDoc Go Gel Imaging System, Denmark). Positive and negative extraction and PCR controls were included in the analysis. A 1 kb plus DNA ladder (catalogue no.: Invitrogen 10787018/10781026, ThermoFisher Scientific, Denmark) was used for size estimation of DNA fragments and classification: type 0 (188 bp), 1 (188 and 549 bp), 2 (188 and 841 bp), or 3 (188 and 361 bp) (Rochat et al. 2017).

### 2.5 Genome Sequencing

#### 2.5.1 DNA Extraction

The bacteria were prepared for sequencing as follows. A starter broth culture (5 mL of TYES‐B) was prepared by the inoculation of material from the −80°C stocks and incubated for 48 h before plating on TYES‐A (15°C, 115 RPM). After 5 days, one colony was picked and transferred into 5 mL TYES‐B and incubated at 15°C (115 RPM) for 72 h. The QIAamp DNA Mini kit (catalogue no.: 51306, Qiagen, Denmark) was then used for DNA isolation. Briefly, bacterial cells (1.5 mL of 72‐h grown cultures) were harvested by centrifugation (5 min at 5000 × g) and incubated at 56°C with 180 μL of ATL buffer and 20 μL of Proteinase K for 3 h (in 1.5 mL DNA LoBind tubes (catalogue no.: 022431021, Eppendorf SE, Germany) that were vortexed during the incubation). To ensure RNA–free material, RNase A (100 mg/mL, 4 μL) was added, mixed by pulse‐vortexing for 15 s, and the material incubated for 2 min at room temperature (15°C–25°C). DNA isolation was then completed following the manufacturer's instructions (modifications of elution step: 100 μL of elution buffer, 5 min incubation). A negative control for the extraction (only buffers and kit components) was included to ensure no cross‐contamination and contamination from kit components. DNA was stored at 4°C overnight and then sent to DNASense ApS (Aalborg, Denmark) for ONT sequencing.

#### 2.5.2 DNA Quality Control

Before shipping, extracted DNA concentration and purity were measured with Qubit 1× dsDNA HS Assay kit (catalogue no.: Q33231, Invitrogen by Thermo Fisher Scientific, USA) on the instrument Fluorometer Qubit 4 (Invitrogen by Thermo Scientific, Singapore) and with the NanoDrop ND‐1000 Spectrophotometer (Saveen Werner, Sweden), and DNA integrity was assessed by gel electrophoresis (30 min) [E‐Gel Agarose Gel with SYBR Safe DNA Gel Stain, 1% (catalogue no.: A42100, Invitrogen by Thermo Fisher Scientific, Denmark) visualized with the instrument Bio‐Rad GelDoc Go Imaging System (Bio‐Rad GelDoc Go Gel Imaging System, Denmark)]. After shipping, DNA quality was again evaluated by DNASense ApS as follows. Gel electrophoresis on Genomic DNA ScreenTapes and using the Tapestation 4200 (Agilent, USA) was used to validate DNA size distribution of a subset of DNA extracts. DNA concentration and purity were measured with the Qubit dsDNA HS Assay kit (Thermo Fisher Scientific, USA) and on the NanoDrop One (Thermo Fisher Scientific, USA).

#### 2.5.3 DNA Sequencing and Data Processing (DNASense Aps)

The sequencing technology was selected for genome analysis based on the recent results obtained by DNASense (Sereika et al. 2022). A native‐barcoded SQK‐LSK114 (Ligation sequencing kit V14) library was prepared according to the manufacturer's protocol, except for minor modifications (Oxford Nanopore Technologies, Oxford, United Kingdom). The library was loaded onto a primed R10.4.1 FLO‐MIN114 flow cell and sequenced on a MinION (mk1B) device with MinKNOW Release 22.07.5 (MinKNOW Core 5.3.0‐rc6‐p2solo). FAST5 data was basecalled using the super accuracy basecalling (SUP) algorithm (Guppy v. 6.3.8). Basic read statistics including the amount of data produced, median read quality and read N50 were assessed with NanoPlot version 1.39.0 (De Coster et al. 2018).

### 2.6 Bioinformatics Analysis

#### 2.6.1 *De Novo* Assembly

Draft *de novo* assemblies were produced with Flye v. 2.9.1‐b1780 (Lin et al. 2016). Draft assemblies were subsequently polished one time with Racon v. 1.5.0 (Vaser et al. 2017) and Minimap2 v. 2.24‐r1122 (Li 2018) and twice with Medaka v. 1.7.2 (Oxford Nanopore Technologies, Oxford, United Kingdom). Assembly graphs and draft assembly coverage were visualized and inspected with Bandage (v. 0.8.1 (Wick et al. 2015)). Each genome assembly was quality‐assessed with CheckM v. 1.1.3 (Parks et al. 2015). Final assembly consensus accuracy was assessed by remapping ONT reads to the polished assemblies using Minimap2 v. 2.31 (Li 2018) followed by residual variant detection using Clair3 v. 2.0.1 (Zheng et al. 2022) with an ONT‐specific model. Residual high‐confidence variants passing predefined quality scores (QUAL ≥ 20) and allele‐frequency thresholds (AF ≥ 0.5) were interpreted as potential remaining consensus errors and used to estimate empirical consensus error rates [error rate = (number of retained residual variants)/(assembly size)] and corresponding Phred‐scaled assembly QV values [QV = −10log_10_(error rate)]. Sample genomes were classified with the GTDB (Genome Taxonomy Database) tool Kit v. 2.1.1 (Chaumeil et al. 2020) against the Genome Taxonomy Database (GTDB) r207_V2 (Parks et al. 2020). Annotation was performed with the NCBI's Prokaryotic Genome Annotation Pipeline (PGAP).

#### 2.6.2 Multi‐Locus Sequence Typing (MLST)

To perform the MLST analysis, the newly sequenced assembled genomes were submitted to the PubMLST database, https://pubmlst.org/organisms/flavobacterium‐psychrophilum (14‐02‐2023) (Fujiwara‐Nagata et al. 2013; Jolley et al. 2018). Following allele type (AT) and sequence type (ST) assignation, clonal complexes (CC) were identified. Briefly, CC are defined as STs groups connected based on the criterion of single locus variant (SLV) (Fujiwara‐Nagata et al. 2013) and the predicted CC founder is defined as the ST with the highest number of SLVs. When an ST is not included in a CC, it is referred to as a singleton. One new allele combination type (AT) was observed in one isolate and therefore submitted to the PubMLST database for ST assignation.

Two isolates (21‐9697‐3A1 and 19‐12880‐80A) presented one nucleotide gap in the *trp*B sequences. To confirm the gap being a sequencing/assembly artefact, PCR amplification of the *trp*B segment and Sanger sequencing of the region were performed as follows. The isolate 20‐1298‐14A G was included as a control since it did not present any gap in the region. DNA was extracted, as for genome sequencing. The 50 μL PCR reaction mixture consisted of 25 μL of the DreamTaq Green PCR Master Mix (2×) (catalogue no. K1081, ThermoFisher Scientific, Denmark), 1 μL of DNA template, the set of *trp*B primers developed by (Nicolas et al. 2008) (final concentration of 0.3 μM) and RNase free water. The PCR reactions were heated for initial denaturation at 94°C for 3 min, followed by 35 cycles of 95°C for 30 s, 52°C for 30 s, 72°C for 2 min and a final extension at 72°C for 8 min. The PCR products were analysed using Invitrogen E‐Gel Double Comb Agarose Gels with SYBR Safe DNA Gel Stain, 2% (catalogue no.: Invitrogen A42348, Fisher Scientific, Denmark) [15 min] and visualized with the instrument Bio‐Rad GelDoc Go Imaging System (Bio‐Rad GelDoc Go Gel Imaging System, Denmark). Positive and negative extraction controls plus a negative PCR control were included in the analysis. A 1 kb plus DNA ladder (catalogue no.: Invitrogen 10787018/10781026, ThermoFisher Scientific, Denmark) was included in the gel. Subsequently, PCR products were purified by using the QIAquick PCR Purification Kit (catalogue no.: 28104, QIAGEN) according to manufacturer's recommendations, their final concentration was measured with the NanoDrop ND‐1000 Spectrophotometer (Saveen Werner, Sweden) and Sanger sequencing was performed by Eurofins Genomics (Ebersberg, Germany). The sequences were analysed using CLC Main Workbench 25 (version 25.0).

#### 2.6.3 Genome Analysis Focused on AMR Determinants

Assembled genomes were mapped against AMR‐related genes previously identified by Castillo et al. (2021) [analysis of 26 *F. psychrophilum* genomes; Rapid Annotation using Subsystem Technology, RAST (Overbeek et al. 2005; Aziz et al. 2008)] and by a literature search focused on the antimicrobials of interest (Vedantam et al. 1998; Enne et al. 2002; Saticioglu et al. 2019; Sánchez‐Osuna et al. 2020). The mapping was performed using Geneious Prime 2023.0.4 [Biomatters development team, Copyright 2005–2023 Biomatters Ltd]. Information about present/absence and nucleotide/amino acid mutations were collected: the American type strain *F. psychrophilum* NCIMB1947 was chosen as reference when harbouring the selected gene. Among the genes related to antimicrobial/toxic compounds resistance identified by Castillo et al. (2021), some were unique to the isolate FPS‐R7 (kidney of rainbow trout isolated in 2017 in Russia). These unique genes were included and sequences from FPS‐R7 used as reference.

The assembled genomes (and plasmids) were also submitted to the Comprehensive Antibiotic Resistance Database's (CARD, card.mcmaster.ca) Resistance Gene Finder (RGI) online software (April 2023 and October 2024) (Alcock et al. 2020, 2023) and to ResFinder 4.1 (April 2023) (Camacho et al. 2009; Bortolaia et al. 2020). The CARD's RGI Finder identifies putative AMR determinants classifying them based on the Perfect/Strict/Loose algorithms [Perfect: perfect matches to the curated sequences and mutations; Strict: unknown variants of known antimicrobial resistance genes (ARGs) present in the database; Loose: more distant homologues of known ARGs, including variants that may not be involved in AMR] (Alcock et al. 2020, 2023). ResFinder identifies ARGs in total or partial DNA bacterial sequences and classifies them with a colour‐based code [Dark green: perfect match, 100% identity and same length of the known ARG in the database; Grey: non‐perfect match, alignment length shorter than ARG in the database (identity < 100%); Light green: non‐perfect match, one/more base variations are present (identity < 100%)] (Camacho et al. 2009; Bortolaia et al. 2020). Alignment of nucleotide/amino acid sequences identified by the database and reference ARGs was performed with Geneious Prime 2023.0.4 [Biomatters development team, Copyright 2005–2023 Biomatters Ltd].

#### 2.6.4 Characterization of Identified *floR* Gene and Its Genomic Location

The identified ARG *floR* was further characterized by submitting the aa sequence to Blastp [Nr database (Title: All non‐redundant GenBank CDS translations + PDB + SwissProt + PIR + PRF excluding environmental samples from WGS projects; Molecule Type: Protein; Update date: 2024/09/11; Number of sequences: 799489739)] (Altschul et al. 1997).

To analyse the genomic location of the *floR* gene, bacterial genomes were submitted to IslandViewer 4 (Bertelli et al. 2017) and recommendations by Langille et al. (2010) were followed (i.e., consideration of predicted genomic islands (GI) by more than one method, region size, presence of mobility genes such as integrases and transposases, GC content). The selected predicted GIs and their flanking regions were manually inspected with Geneious Prime 2025.2.2 [Geneious development team, Copyright 2005–2025 GraphPad Software LLC d.b.a Geneious]. The presence of ribosomal genes at one end and the identification of integrase genes/tRNA in the other were used as elements to define the borders of the predicted GI. The whole collection of genomes included in the study was inspected for the corresponding GI. When identified, genomic regions were extracted and aligned with Progressive Mauve (Darling et al. 2010) in Geneious Prime 2025.2.2.

#### 2.6.5 Pangenome and Phylogenetic Analysis

To assess the genomic content (e.g., presence of unique genes) among isolates with similar antimicrobial‐resistant profiles, a pangenome analysis was performed. Assembled genomes were at first annotated with Prokka v.1.14.6 (Seemann 2014) and then a pangenome analysis was performed with Roary v.3.13.0 with default parameters (BlastP identity cutoff of ≥ 95%) (Page et al. 2015). Based on the core genes alignment (PRANK codon aware alignment), a maximum likelihood tree was generated with FastTree 2.0 (Price et al. 2010). Computational analysis was performed on the GenomeDK cluster (Aarhus University, Denmark). Phandango (Hadfield et al. 2018), GraphPad Prism v.10.4.1 for Windows (GraphPad Software, Boston, Massachusetts USA, www.graphpad.com) and Inkscape v.1.4.2 (2025 Inkscape Developers, www.inkscape.org) were used for the visualization of the results. Unique genes to each isolate were inspected manually and categorized based on the UniProt database (The UniProt Consortium 2025).

## 3 Results

Antimicrobial susceptibility of *Flavobacterium psychrophilum* isolates (Table 1) was assessed focusing on antimicrobials used in Danish aquaculture (FLO, OXO, TRS, AMO, OXY) (Figure 1). Figure 2 summarizes the results of the phenotypic and genotypic characterization. Epidemiological background (MLST), serotype, virulence (median lethal dose (LD_50_)) and genomic basic features (genome size and presence of plasmids) are presented in Figure 2A, while Figure 2B provides an overview of the results of the antimicrobial susceptibility testing (AST) and the genome analysis focused on AMR determinants. Detailed AST results can be found in Table 2 for disc diffusion assays (A) and for broth micro‐dilution method (B). The bacterial genomes were sequenced (Tables S1 and S2: sequencing and assembly statics and genomes accession numbers) and then analysed for genes involved in resistance to antimicrobials and toxic compounds (Figure 2B and Figure S1).

**FIGURE 2:**
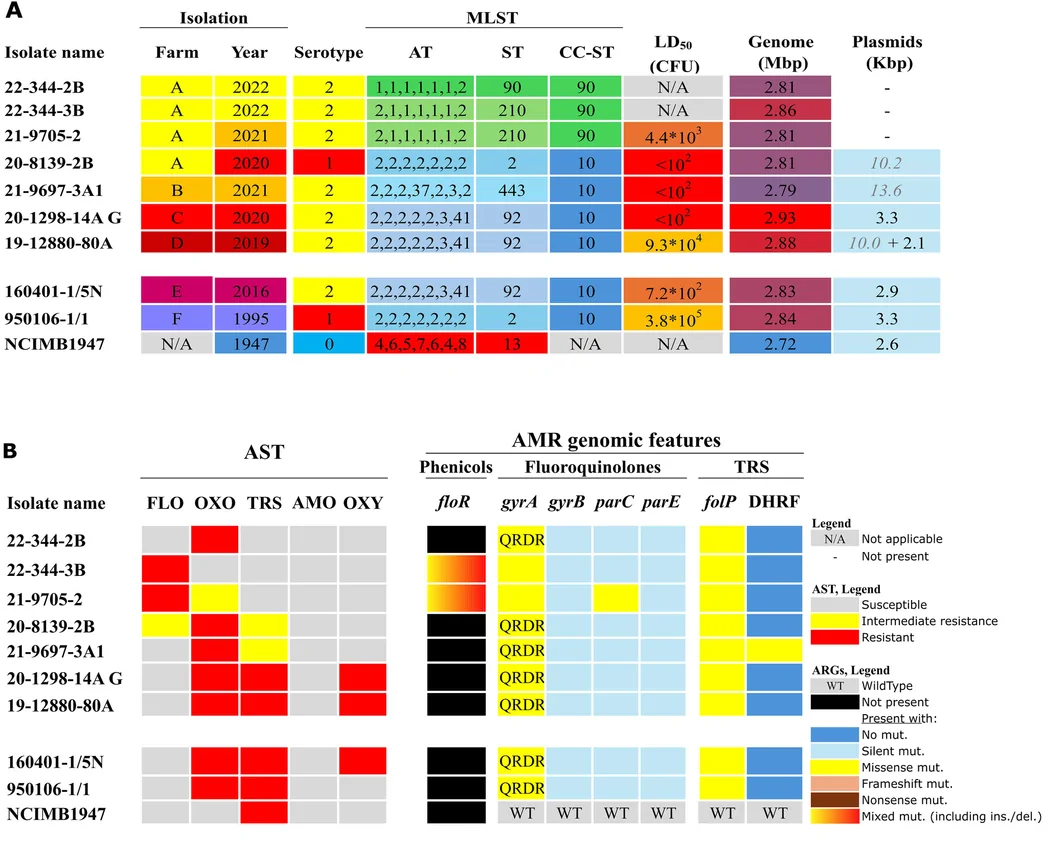
Phenotypic and genotypic features of the selected panel of *F. psychrophilum* isolates. In (A), isolation information (farm and year), serotype, epidemiological background (MLST), in vivo virulence estimation by median lethal dose (LD_50_) are presented together with basic genomic information (genome size and plasmids). Variations in the tested parameters are colour coded. Genome sizes are represented on a gradient from blue (smallest) to red (largest). Unusual large plasmids are indicated in italics and in grey as they could be an artefact of ONT seq. and, their sizes were not confirmed experimentally. In (B), results from the AST of antimicrobials relevant to Danish aquaculture alongside with the main results of the genome analysis targeting AMR determinants are summarized. For the AST, resistant/NWT and susceptible/WT phenotypes are highlighted in red and in grey, respectively. Intermediate resistance (values in the vicinity of resistance/NWT thresholds) observed by either one of the AST methods (disc diffusion or micro‐dilution AST) is highlighted in yellow. Details on AST can be found in Table 2. Regarding the AMR genomic features analysis, mutations compared to genes in the American wildtype (WT) NCIMB1947 are illustrated. For the gene identified by ResFinder/CARD‐RGI Finder, mutations compared to the databases sequences are presented. A more comprehensive overview of genes associated with resistance to antimicrobials and to toxic compounds included in the analysis with further details about types of mutations and loci numbers can be found in Figure S1. AMO, amoxicillin; AMR, Antimicrobial Resistance; AST, Antimicrobial Susceptibility Testing; AT, Allele type (order: *AtpA, dnaK, fumC, gyrB, murG, trpB, tuf*); CC‐ST, clonal complex; CFU, Colony Forming Unit; FLO, florfenicol; MLST, Multi‐Locus Sequence Typing; N/A, not applicable (not performed); OXO, oxolinic acid; OXY, oxytetracycline; QRDR, Quinolone Resistance Determining Region; ST, sequence type; TRS, trimethoprim/sulfamethoxazole.

**TABLE 2:** AST: Disc diffusion assay (A) and broth micro‐dilution method (B) determinations.

A					
Isolate name	Disc diffusion assay (diameter of inhibition zone, mm)^a^				
	FLO	OXO	TRS	AMO	OXY
**22‐344‐2B**	> 70	**7**	60	60	70
**22‐344‐3B**	**8**	40	36	64	60
**21‐9705‐2**	**7**	**30**	60	70	70
**20‐8139‐2B**	40	**7**	**25**	70	60
**21‐9697‐3A1**	55	**8**	**25**	60	50
**20‐1298‐14A G**	60	**6**	**7**	70	**30**
**19‐12880‐80A**	60	**6**	**7**	60	**30**
160401‐1/5N	60	**7**	**7**	70	**32**
950106‐1/1	60	**7**	**7**	65	62
NCIMB1947	60	40	**7**	70	60

B											
Isolate name	MIC values (μg mL^−1^)^b^									Growth controls	
	Phenicols	Quinolones		Sulfonamides + comb.		Lactams			Aminoglycoside		
	FLO	ENR	OXO	SME	TRS	AMP	CTZ	MER	GEN	Pos.	Neg.
**22‐344‐2B**	1	**> 0.25**	**> 1**	8	0.5/9.5	0.06	1	< 0.008	0.5	+	−
**22‐344‐3B**	**> 16**	**0.06**	0.12	8	0.5/9.5	0.06	1	< 0.008	0.5	+	−
**21‐9705‐2**	**> 16**	**0.06**	0.25	8	0.5/9.5	0.06	1	< 0.008	0.5	+	−
**20‐8139‐2B**	**4**	**0.25**	**> 1**	8	1/19	0.06	0.5	< 0.008	0.5	+	−
**21‐9697‐3A1**	2	**> 0.25**	**> 1**	8	0.5/9.5	0.06	0.25	< 0.008	2	+	−
**20‐1298‐14A G**	1	**> 0.25**	**> 1**	8	0.5/9.5	0.06	0.5	< 0.008	1	+	−
**19‐12880‐80A**	0.5	**> 0.25**	**> 1**	16	1/19	0.06	0.5	< 0.008	1	+	−
160401‐1/5N	0.5	**> 0.25**	**> 1**	8	0.5/9.5	0.06	0.5	< 0.008	0.5	+	−
950106‐1/1	1	**> 0.25**	**> 1**	8	0.5/9.5	0.06	0.5	< 0.008	1	+	−
NCIMB1947	0.5	**0.06**	0.12	8	0.5/9.5	0.12	0.5	< 0.008	2	+	−
*A. salmonicida* subsp. *salmonicida* ATCC 33658	**0.5**	**0.03**	**0.03**	512	**0.25/4.75**	**0.12**	< 0.03	0.03	< 0.06	+	−
*E. coli* ATCC 25922	**4**	0.002	**0.06**	16	**0.12/2.38**	**8**	0.12	< 0.008	< 0.06	+	−

*Note:* Resistance/reduced susceptibility was determined according to routinely used interpretive criteria for disc diffusion assays [6] (A) and according to established epidemiological cut‐off values (ECVs) for micro‐dilution assays [15] (B). In B, acceptable QC ranges are provided for AMP, FLO, OXO, OXY and TRS (1/19) for both QC bacteria while only for *A. salmonicida* sub*sp. salmonicida* ATCC33658 for ENR (in bold and blue). For TRS and OXY, we observed not satisfactory MIC QC for one QC bacterium in TRS (in bold and red) and for both QC bacteria in OXY. Values for OXY were so excluded.

Abbreviations: AMO, amoxicillin; AMP, ampicillin; CTZ, ceftazidime; ENR, enrofloxacin; FLO, florfenicol; GEN, gentamicin; MER, meropenem; NWT, Non‐wild type; OXO, oxolinic acid; OXY, oxytetracycline; QC, Quality control; SME, sulfamethoxazole; TRS, sulfamethoxazole/trimethoprim.

^a^ Values above resistance threshold are in bold and highlighted in red: to OXO when diameter ≤ 30 mm; to TRS when diameter ≤ 30 mm; to OXY when diameter ≤ 42 mm; to FLO when diameter ≤ 30 mm; to AMO when diameter ≤ 31 mm [6]. Discs are 6 mm in diameter.

^b^ NWT patterns for FLO, ENR, OXO are in bold and highlighted in red [15]. Deviation from CLSI guidelines VET03: TYES‐B instead of diluted CAMHB for inoculum preparation due to growth issues.

### 3.1 Bacterial Isolates

*Flavobacterium psychrophilum* isolates included in this study originated from Danish freshwater farms and were isolated in the period 2019–2022 from the internal organs (spleen and kidney) of rainbow trout (Table 1). Four isolates originated from one RAS farm in the period 2020–2022 (farm A). Specifically, we received samples for AST in relation to RTFS outbreaks associated with 10–16 days of florfenicol ineffective treatment in 2020 and 2021 (20‐8139‐2B and 21‐9705‐2). In 2022, further samplings from the same farm were performed in an outbreak case before initiating antibiotic treatment to support the correct use of antimicrobials (isolates: 22‐344‐2B and 22‐344‐3B; from the kidneys of two different fish). These isolations performed in connection to diagnostic cases were included in this analysis because of their unique and previously unobserved antimicrobial resistance profile.

The remaining three isolates originated from three other farms (farm B, C and D) in relation to recent research projects focused on the optimization of infection methods in fish. The isolate 21‐9697‐3A1 originated from an outbreak case in RAS farm B while the isolate 20‐1298‐14A G was retrieved from a fish with non‐apparent clinical signs within a batch suffering from an infection from *Yersinia ruckeri* (Farm C, also RAS). Finally, the isolate 19‐12880‐80A originated from naïve fish in cohabitation with fish transported from a fish farm to our facilities, which then developed skin lesions and thus it was sampled. The well‐characterized Danish isolates *F. psychrophilum* 950106‐1/1 and 160401‐1/5N were included in the study together with the American strain NCIMB1947 as references.

### 3.2 Antimicrobial Susceptibility Testing (AST)

FLO resistance/reduced susceptibility was detected in two isolates (farm A; isolation period 2021–2022) with an inhibition diameter of 7 mm and MIC values > 16 μg mL^−1^ (22‐344‐3B and 21‐9705‐2) (Table 2). The isolate 20‐8139‐2B, also from farm A (2020), showed a reduced susceptibility to FLO in the disc diffusion assay without crossing the established threshold for resistance, and it was characterized by a MIC value of 4 μg mL^−1^ (just above the threshold for NWT categorization). From the same diagnostic case where we isolated the FLO‐resistant 22‐344‐3B, we could isolate *F. psychrophilum* FLO‐susceptible (22‐344‐2B) (from a different fish). These two isolates were also characterized by an opposite susceptibility profile in relation to OXO. All the other isolates were resistant or characterized by a reduced susceptibility to OXO (inhibition diameter < or = 30 mm; MIC values > 1 μg mL^−1^). Resistance or reduced susceptibility to TRS (inhibition diameter < or = 30 mm) was detected in most isolates except 22‐344‐2B, 22‐344‐3B and 21‐9705‐2. For TRS, MIC values were towards the high range of concentrations i.e., they were all equal to 0.5/9.5 μg mL^−1^ except 20‐8139‐2B and 19‐12880‐80A whose MIC values were equal to 1/19 μg mL^−1^. For SME, MIC values were all equal to 8 μg mL^−1^ except 19‐12880‐80A whose MIC value was 16 μg mL^−1^.

No resistance/reduced susceptibility was detected in relation to AMO. Only three isolates (20‐1298‐14G, 19‐12880‐80A and 160401‐1/5N) were characterized by a decreased susceptibility to OXY (inhibition diameter ≤ 42 mm; broth micro‐dilution test was excluded for this antimicrobial as MIC QC values were outside the recommended ranges—data not shown). For the other lactams tested with the broth micro‐dilution method, MIC values were all in the low range of tested concentrations: MIC AMP = 0.06–0.12 μg mL^−1^, MIC CTZ = 0.25–1 μg mL^−1^ and MIC MER < 0.008 μg mL^−1^. All the isolates were categorized as NWT in case of enrofloxacin (ENR). Finally, MIC values towards the aminoglycoside GEN ranged from 0.25 to 2 μg mL^−1^.

### 3.3 DNA Sequencing and *De Novo* Assembly

The extracted DNA was subjected to quality control, DNA library preparation and sequencing using Oxford Nanopore R10.4.1 pore chemistry. The quality score was *on par* with the raw read accuracy supported by the current kit chemistry (V14 kit), flow cell (R10.4.1), and basecalling algorithms. All samples generated more than 100× genome data depth or 300 megabases (Mb) (the genome of *F. psychrophilum* is ~3.0 Mb) which is the R10.4.1 data coverage recommended by DNASense for downstream *de novo* assembly and genome polishing workflows [average data: 658 Mbp; average reads no.: 120833; average read length N50: 7850 bp; q‐score ≥ 19.0] (Table S1). All samples generated fully closed bacterial genomes that were supported by more than the minimum recommended data depth (Sereika et al. 2022). The *de novo* assembled genomes were subsequently classified as *F. psychrophilum* using the Genome Taxonomy Database (GTDB) and a total of 0 out of the 7 (0%) samples were contaminated above a 5% contamination threshold adopted MIMAG criteria (Bowers et al. 2017). Basic genome assembly statistics and accession numbers of the newly sequenced *F. psychrophilum* isolates can be found in Table S2. The genome size of the sequenced bacteria varied between 2.79 and 2.93 Mbp with a GC content between 32.5% and 32.8%. One or two plasmids were found in all sequenced isolates except 22‐344‐2B, 22‐344‐3B and 21‐9705‐2. The commonly found 3.3 and 2.2 Kbp Fp plasmids were identified in 20‐1298‐14A G and 19‐12880‐80A, respectively. Three large plasmids resulted from the assembly of 21‐9697‐3A1 (circa 13 Kbp) and, of 20‐8169‐2B and 19‐12880‐80A (circa 10 Kbp). These unusual large plasmids seemed to be multiple tandem copies of the same plasmid sequence, thus likely concatemer assembly artefacts. Because the aim of the project was AMR determinants characterization, large plasmids sizes were not verified experimentally.

### 3.4 Genome Analysis Focused on AMR Determinants

An overview of the analysed genomic AMR determinants is shown in Figure 2 and Figure S1.

#### 3.4.1 Output of ResFinder and the CARD's RGI Finder

The CARD's RGI Finder did not identify any antimicrobial resistance gene (ARG) with a perfect match, but one with a strict match (very low identity) and several (113–123) with a loose match (See File S1 to navigate the RGI Finder output). Among the loose match results, *floR* was the only gene with 99% identity to the database, and the same gene was also identified by ResFinder (grey match) (*see next paragraph ‘Phenicols’*). All the other DNA sequences with a loose match were characterized by identity < 70%. No ARGs were found in plasmids.

#### 3.4.2 Phenicols

The chloramphenicol/florfenicol efflux Major Facilitator Superfamily (MFS) transporter gene, *floR*, was identified by ResFinder and the CARD's RGI Finder with non‐perfect matches (grey match and loose match, respectively) with the databases sequences i.e., identity (97%–99%) and gene length (Table 3A) in two isolates: *F. psychrophilum* 21‐9705‐2 (locus number ACFUFK_06145) and 22‐344‐3B (locus number: ACFUFJ_07055). A Blastp search (01‐05‐2023 and 11‐09‐2024) showed the high similarity with *floR* proteins in *Riemerella anatipestifer* and *Chryseobacterium muglaense* (Table 3B). The multiple alignment of aa sequences of the newly‐identified *flo*R genes, the results of blastp search [99% query cover and 0.0 *E* value], the reference sequences of ResFinder and CARD databases (404 aa) and previously identified *floR* genes in *Chryseobacterium* sp. and *F. psychrophilum* (Verner‐Jeffreys et al. 2017; Saticioglu et al. 2019) highlighted the longer length of the newly observed proteins (413 aa, Figure 3). Furthermore, a ~150 kb genomic island (GI) harbouring the *floR* gene (*floR* GC content: 57.4%) was identified in 21‐9705‐2 (average genome GC content: 32.8%) and 22‐344‐3B (average genome GC content: 32.7%) together with a series of transposase genes, insertion elements and genes related to resistance to antimicrobials and toxic compounds. Figure 4 visualizes this GI among the whole panel of selected isolates, where other antimicrobial/toxic compounds resistance genes are also shown together with conserved regions flanking the genomic area. An integrase gene, conserved among the whole panel of selected isolates, was found in proximity to *floR* suggesting its potential role in the acquisition of the new ARG in the *floR*‐harbouring genomes. The raw results obtained by IslandViewer 4 are presented in Figure S2.

**TABLE 3:** Identification and characterization of *flo*R.

A								
ResFinder								
Isolate	ARG [colour criteria]	Identity (%)	Alignment length/gene length	Coverage	Contig	Position in contig	Phenotype	Accession no.
22‐344‐3B	floR [grey]	97.93	1210/1215	99.5885	1	1565095…1566304	Phenicol resistance	AF118107
21‐9705‐2	floR [grey]	97.93	1210/1215	99.5885	1	1391993…1393202	Phenicol resistance	AF118107

CARD's RGI Finder								
Isolate	ARO term [RGI criteria]	% Identity matching region	% Length of reference sequence	Pass bitscore/best hit bitscore	Contig	Position in contig	Drug class—AMR gene family	ARO accession no.
22‐344‐3B	floR [Loose]	99.0	102.23	750/742.7	1	1565094…1566335	Phenicol antibiotic‐MFS antibiotic efflux pump	3002705
21‐9705‐2	floR [Loose]	99.0	102.23	750/742.7	1	1391992…1393233		3002705

B								
Description	Scientific name	Max score	Total score	Query cover (%)	*E* value	Per. ident (%)	Acc. Len	Accession
Chloramphenicol/florfenicol efflux MFS transporter FloR	*Riemerella anatipestifer*	800	800	100	0.0	99.52	413	WP_154469519.1
Chloramphenicol/florfenicol efflux MFS transporter FloR	*Chryseobacterium muglaense*	798	798	100	0.0	99.27	413	WP_191178984.1
Chloramphenicol/florfenicol efflux MFS transporter FloR	*Riemerella anatipestifer*	797	797	100	0.0	99.03	413	WP_214192577.1
Chloramphenicol/florfenicol efflux MFS transporter FloR	*Riemerella anatipestifer*	796	796	100	0.0	98.79	413	WP_154138268.1
Chloramphenicol/florfenicol efflux MFS transporter FloR	*Halopseudomonas bauzanensis*	777	777	97	0.0	99.25	404	WP_285261509.1
Chloramphenicol/florfenicol efflux MFS transporter FloR	*Enterobacteriaceae*	776	776	97	0.0	99.50	404	WP_180847137.1
Chloramphenicol/florfenicol efflux MFS transporter FloR	*Escherichia coli*	775	775	97	0.0	99.00	404	WP_136763179.1
Chloramphenicol/florfenicol efflux MFS transporter FloR	*Pseudomonas yangonensis*	775	775	97	0.0	99.01	404	WP_161897966.1
Chloramphenicol/florfenicol efflux MFS transporter FloR	*Escherichia coli*	775	775	97	0.0	99.25	404	WP_099145544.1
Chloramphenicol/florfenicol efflux MFS transporter FloR	*Enterobacteriaceae*	775	775	97	0.0	99.25	404	WP_039026029.1

*Note:* (A) Results of *floR* identification by ResFinder and CARD's RGI Finder in *F. psychrophilum* 21‐9705‐2 and 22‐344‐3B; (B) Results of *floR* Blastp search. The first 10 best matches are presented and the proteins used for the multiple alignment in Figure 4 are highlighted in yellow.

**FIGURE 3:**
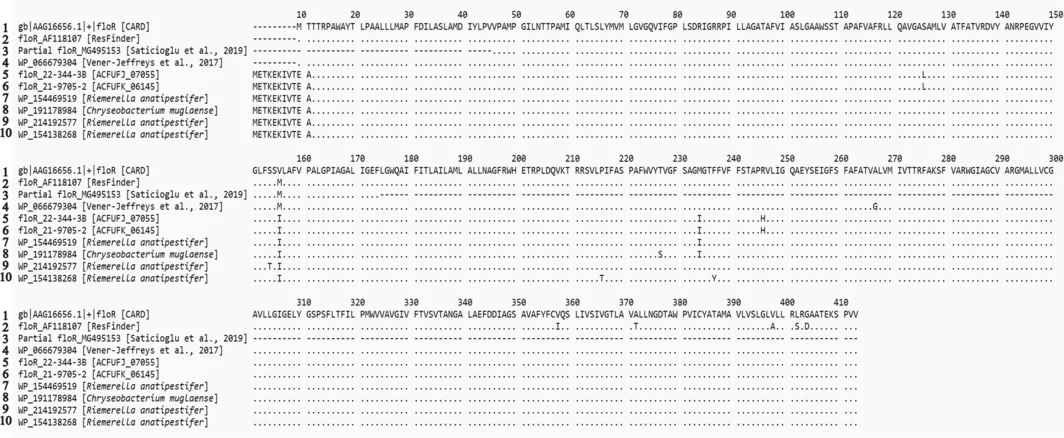
Multiple alignment of *floR* genes translations. (1) CARD/RGI database (ARO: 3002705); (2) ResFinder database (best match); (3) partial *floR* identified in a florfenicol‐susceptible *F. psychrophilum* isolate in Turkey by Saticioglu et al. (2019) [NCBI locus: MG495153]; (4) *floR* identified by Verner‐Jeffreys et al. (2017) in *Chryseobacterium* sp. and not strongly related to florfenicol resistance [NCBI locus: WP_066679304]; (5) putative *floR* identified in 22‐344‐3B; (6) putative *floR* identified in 21‐9705‐2; 7–10, sequences from blastp of putative FloR proteins [100% query cover and 0.0 *E* value]. Multiple alignment was performed with Geneoius Prime. All disagreements are highlighted with the corresponding aa mismatch. In case of similarity, dots are used. The stop codon is not included in the sequences.

**FIGURE 4:**
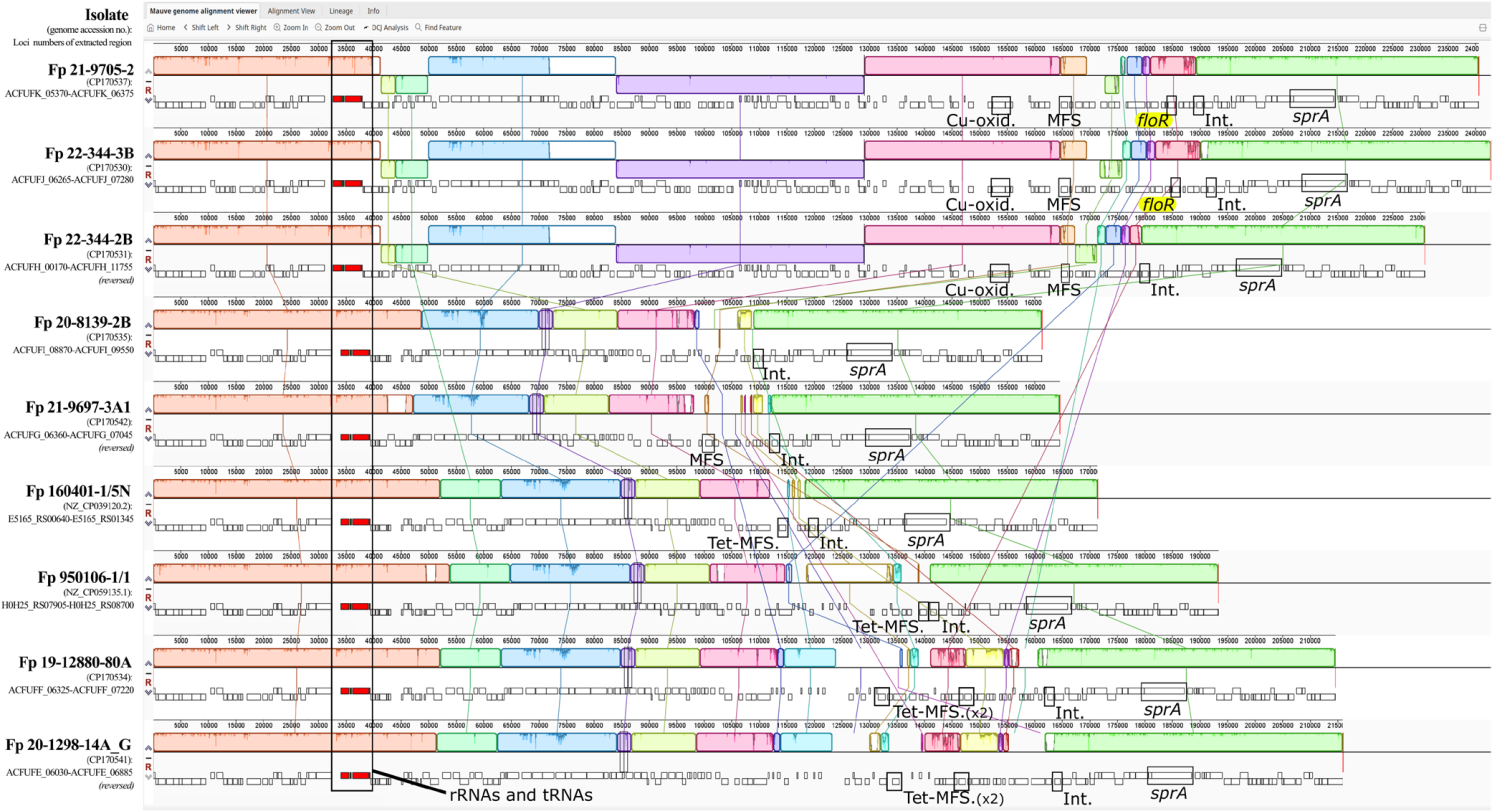
Visualization of Genomic Island (GI) harbouring *floR* and other resistance/mobility genes together with conserved flanking regions. A large GI was predicted by IslandViewer 4 by both IslandPath‐DIMOB and SIGI‐HMM in the proximity of the *floR* gene in 21‐9705‐2 and 22‐344‐3B (Figure S2). The predicted GIs and flanking regions of *floR*‐harbouring genomes as well as the corresponding areas in the other isolates included in the study were extracted, aligned with Progressive Mauve and manually inspected. Locally collinear blocks (LCB) are presented, each with its unique colour. Isolates names and corresponding loci numbers of extracted regions are presented left to each genomic area. The presence of ribosomal genes at one end (in red) and the identification of a conserved integrase gene in the other (indicated by *Int*.) were used as elements to define the border of the predicted GI. Examples of highly conserved genes between genomes are indicated such as rRNAs, tRNAs as well as *sprA* (component of Type‐9 Secretion System, T9SS). Mobility genes such as transposases and integrases were found as well as genes related to resistance to antimicrobials and other toxic compounds, such as *floR* (in yellow), MDR MFS transporter (indicated by ‘MFS’) and multicopper oxidase (indicated by ‘Cu‐oxid.’) are indicated. While analysing the regions, the presence of a tetracycline MFS transporter (indicated by ‘Tet‐MFS’) was also identified in the Danish references strains as well as in two new isolates with two identical copies: 19‐12880‐80A and 20‐1298‐14A G.

#### 3.4.3 Quinolones/Fluoroquinolones

Nucleotide alignment of *gyrA* and *gyrB* (DNA gyrase) and of *parC* and *parE* (DNA topoisomerase IV) was performed (Figure 5, WT: *F. psychrophilum* NCIMB1947). Silent mutations were observed in all the isolates for all four genes. Missense mutations were observed in *parC* in the isolate 21‐9705‐2 [pos. 340, from Aspartic acid to Asparagine] and in *gyrA* in all the isolates. In *gyrA*, missense mutations were observed at aa position 82 in the OXO resistant isolates [from Threonine (WT) to Valine (22‐344‐2B, 20‐1298‐14A G, 19‐12880‐80A and 160401‐1/5N), Alanine (20‐8139‐2B, 21‐9697‐3A1) or Arginine (950106‐1/1)] and at position 481 in 22‐344‐3B and 21‐9705‐2 [from Arginine to Cysteine].

**FIGURE 5:**
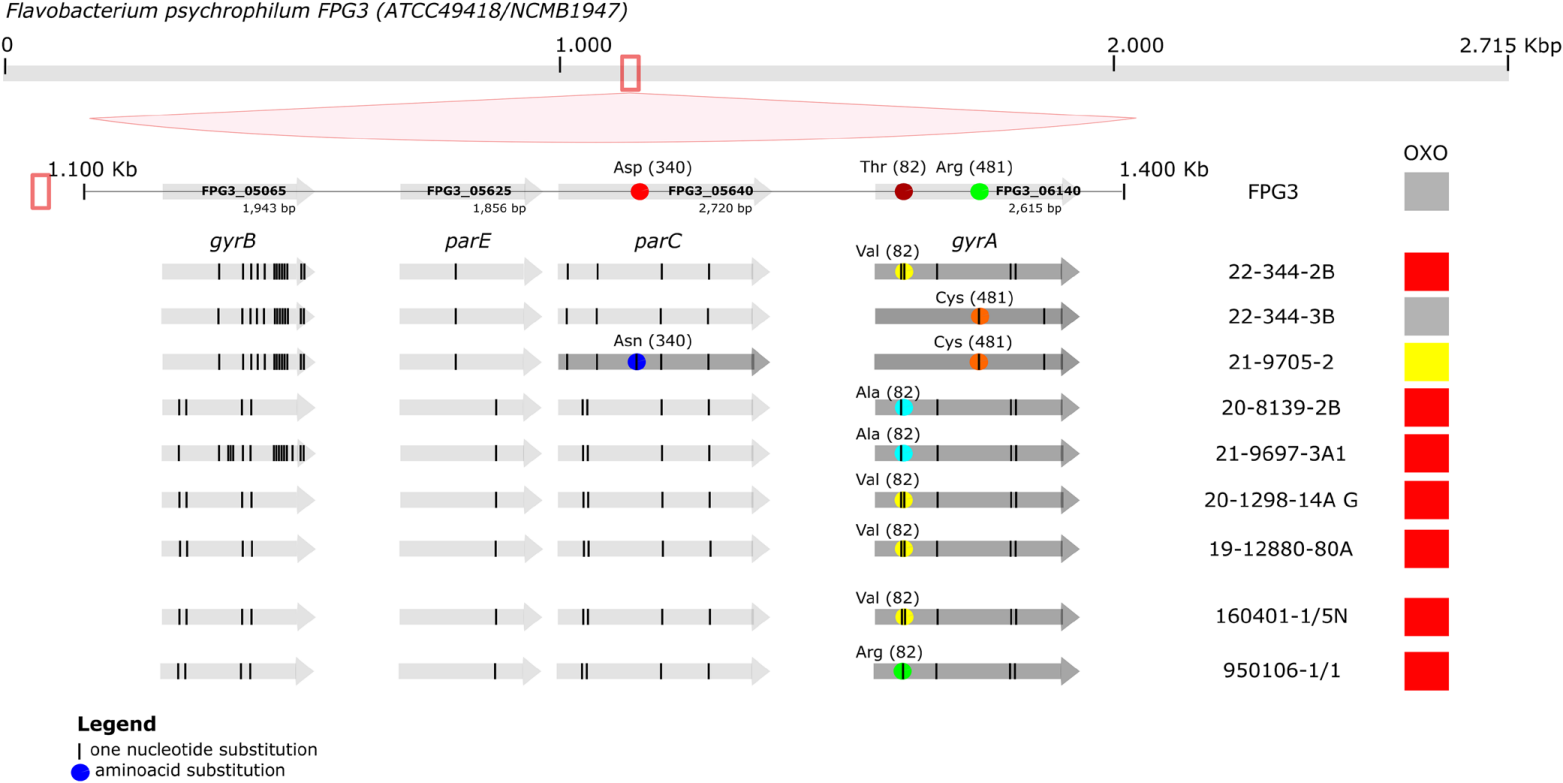
Schematic representation of genes associated with oxolinic acid (OXO) resistance. Since one of the mechanisms of quinolone resistance (QR) is the alteration of the quinolone target enzymes, DNA gyrase (*gyrA* and *gyrB*) and DNA Topoisomerase IV (*parC* and *parE*) were inspected. *F. psychrophilum* NCMB1947 (FPG3) was used as reference for the alignments. Nucleic acid substitutions are indicated by the black lines on the genes, and, in case of missense mutations, the amino acid substitution is represented by a coloured dot (different colour for different aa; the aa position is indicated in the parenthesis). The loci numbers of genes of the reference genome are illustrated while for the other isolates, loci numbers can be found in Figure S1. To the right, the in vitro susceptibility profile of each isolate towards OXO is presented: Susceptible/WT phenotype (grey), resistant/NWT phenotype (red) and intermediate resistance (yellow). Intermediate resistance represents cases where obtained values by either one of the AST methods (disc diffusion or broth micro‐dilution AST) were in the vicinity of resistance/NWT thresholds. Details on AST can be found in Table 2.

#### 3.4.4 Sulfamethoxazole/Trimethoprim

The dihydropteroate synthase (DHPS or *folP*) and the dihydrofolate reductase (DHFR) coding genes were aligned together with sequences from the type strain *F. psychrophilum* NCIMB 1947/ATCC49418 (FPG3) (Figure S3). Missense mutations were observed in the DHPS genes of all the isolates. However, differences in the number and location of the mutations were observed. For the DHFR coding gene, one silent mutation and one missense mutation were observed in 21‐9697‐3A1.

#### 3.4.5 Other Determinants of Resistance Towards Antimicrobials and Toxic Compounds

Putative genes related to multidrug resistance (MDR) and to resistance to toxic compounds previously identified for *F. psychrophilum* (Castillo et al. 2021) were taken into consideration (genes and loci numbers in Figure S1). Among the first category, an MFS transporter (Major Facilitator Superfamily) was found without any mutation in four isolates, three originating from farm A (22‐344‐2B; 22‐344‐3B; 21‐9705‐2) and 21‐9697‐3A1. A blastp search (March 2023) was conducted to gain some insight into the presence of this transporter among other strains and the first 10 best matches are presented in Table S3, showing its presence in isolates originating from *O. mykiss* in Russia, Chile and Czech Republic [100% query cover, 100% identity and 0.0 *E* value] but also from other fish species such as grayling ( *Thymallus thymallus* ) and ayu ( *Plecoglossus altivelis* ) [99% query cover, 85%–99% identity and 0.0 *E* value]. Interestingly, this MFS transporter is in the same GI as the *flo*R gene (Figure 4). A Resistance Nodulation Division family (RND) multidrug efflux transporter was identified in all the isolates with missense mutation except 22‐344‐2B where a nonsense mutation was identified. Among the genes analysed for resistance to toxic compounds, a multicopper oxidase was unique to three isolates from farm A (22‐344‐2B; 22‐344‐3B; 21‐9705‐2) and it was also located in the same GI as the *floR* gene (Figure 4).

### 3.5 Other Characterizations: in vivo Virulence, Serotype and Epidemiological Background

#### 3.5.1 Median Lethal Dose (LD_50_ )

Five of the new isolates were tested in animal experiments for LD_50_ determinations: Figure 2 and Figure S4. Results showed that the LD_50_ of the five isolates ranged from values off scale, so below 10^2^ CFU (20‐8139‐2B, 20‐1298‐14A, 21‐9697‐3A1) to 4.4 × 10^3^ CFU (21‐9705‐2) and 9.3 × 10^4^ CFU (19‐12880‐80A). Danish reference strains were included in the fish trials as controls and the LD_50_ values were 7.2 × 10^2^ CFU for 160401‐1/5N and 3.8 × 10^5^ CFU for 950106‐1/1. For two isolates (20‐198‐14A and 20‐8139‐2B), events of mortality in the TYES‐injected controls were observed (1 and 3 events respectively) around 11–20 days post infection. All dead and moribund fish were positive for *F. psychrophilum* .

#### 3.5.2 Serotype

All bacterial isolates were subjected to serotype determination. Except 20‐8139‐2B (serotype 1, equivalent to Fd serotype (Lorenzen and Olesen 1997)), all the other *F. psychrophilum* isolates belonged to serotype 2 (equivalent to serotype Th (Lorenzen and Olesen 1997)) (Figure 2).

#### 3.5.3 MLST

Genome‐based MLST showed the presence of isolates belonging to unrecorded sequence types (ST) in Denmark (Figure 2). Indeed, ST90 and ST210, belonging to the clonal complex CC‐ST90, were observed in farm A for the outbreak cases in 2021 and 2022 (22‐344‐2B, 22‐344‐3B and 21‐9705‐2) associated with in vitro and/or in the field reduced susceptibility to florfenicol. In the same farm, the isolate 20‐8139‐2B originating from a previous RTFS outbreak belonged to a different clonal complex, CC‐ST10 (ST2). The other three isolates from farm B, C and D belonged to CC‐ST10 as well (ST92 and ST443). ST443 was newly identified for 21‐9697‐3A1 with the following alleles profile: 2, 2, 2, 37, 2, 3, 2.

### 3.6 Pangenome and Phylogenetic Analysis

To assess the genomic content (e.g., presence of unique genes) among isolates with similar antimicrobial‐resistant profiles, a pangenome analysis was performed. The results of this analysis identified 3475 genes families, divided into 1959 core genes (present in 99%–100% of isolates) and 1516 accessory genes (present in 0%–99%), further categorized into soft core genes (present in 95%–99%; 0 genes), shell genes (present in 15%–95%; 1044 genes) and cloud genes (present in 0%–15%; 472 genes) (Figure 6A). Unique genes to each isolate (Figure 6B) were inspected manually (data not shown). Even if most unique genes were categorized as hypothetical proteins followed by genes involved in metabolic processes, putative genes coding for MDR efflux pumps, so involved in transport/detoxification, were found in 22‐344‐2B and 22‐344‐3B. Clustering of isolates belonging to the same clonal complex was observed with similar accessory genomes (Figure 6C). Shared accessory genes between different CC‐ST were observed for 20‐1298‐14A G and 22‐344‐3B.

**FIGURE 6:**
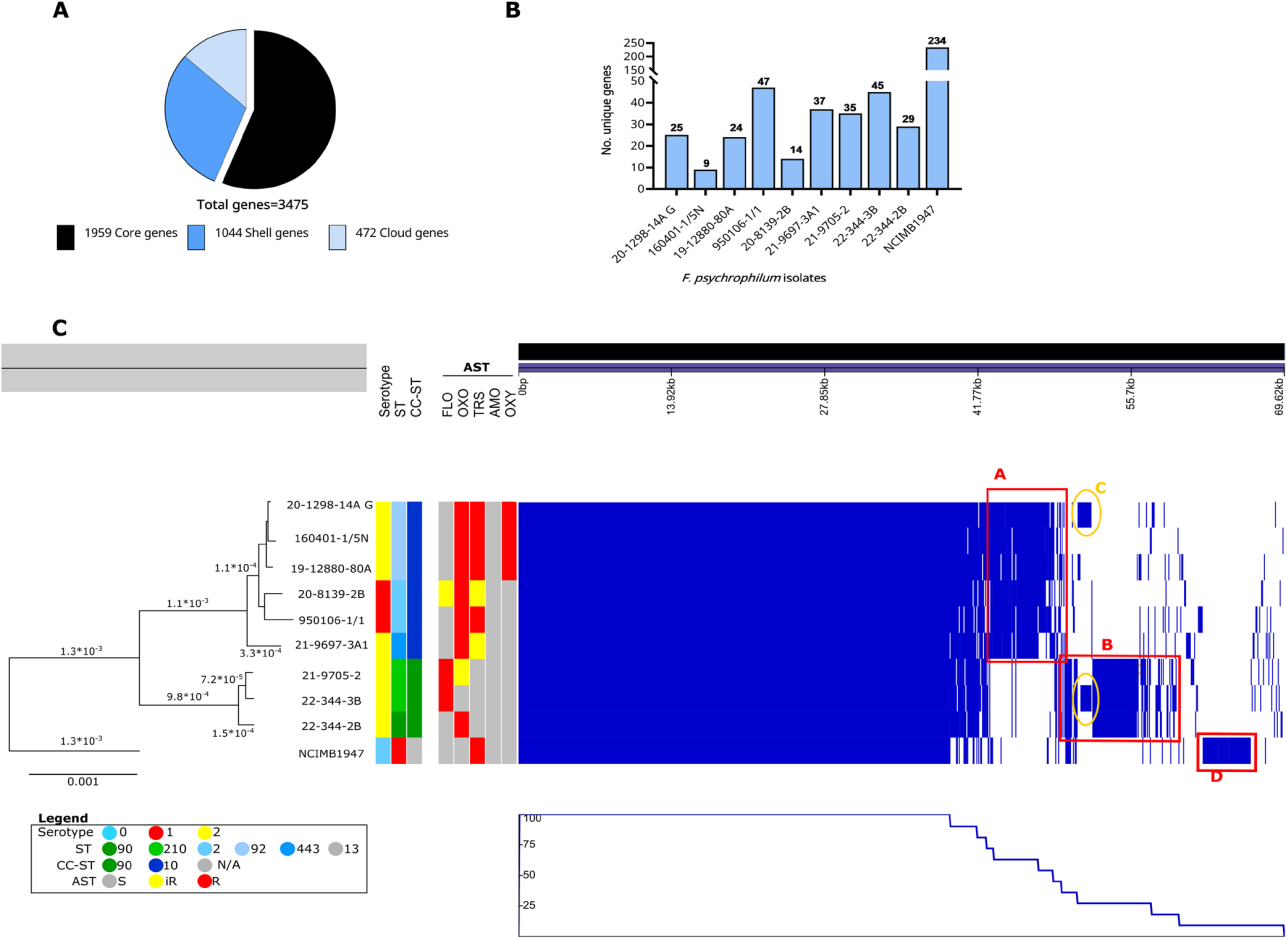
Pangenome and core‐genome phylogenetic analysis. (A) Overview of core (in 99%–100% of the isolates) and accessory genes (present in 0%–99%) among selected panel of isolates (Roary: BlastP identity cutoff of ≥ 95%). Accessory genes are further categorized into soft core genes (present in 95%–99%; 0 genes), shell genes (present in 15%–95%; 1044 genes) and cloud genes (present in 0%–15%; 472 genes). (B) Number of unique genes per isolate. (C) Visualization of pangenome (core and accessory genes), phylogenetic relationship between isolates (maximum likelihood tree with FastTree 2.0 based on core genes PRANK codon aware alignment) and metadata (serotype, epidemiological background, summary of AST). Substitutions per site are indicated for main tree branches. Clustering of isolates belonging to the same CC‐ST is observed and similar accessory genomes are highlighted by red squares (same CC‐ST) and yellow circles (different CC‐ST). AMO, amoxicillin; AST, antimicrobial susceptibility testing (S: Susceptible; iR: Intermediate resistance; R: Resistant); CC‐ST, clonal complex; FLO, florfenicol; N/A, not applicable; OXO, oxolinic acid; OXY, oxytetracycline; ST, sequence type; TRS, trimethoprim/sulfamethoxazole.

Among isolates with reduced susceptibility to OXY, 20‐1298‐14A G and 19‐12880‐80A harboured two exact copies of a putative Tet MFS transport, which were in the same GI as *floR*. The same gene (one copy) was also found in the two Danish reference strains (one with reduced susceptibility, 160401‐1/5N, and one susceptible to OXY, 950106‐1/1), also in this GI.

No unique genes to TRS resistant isolates (or with reduced susceptibility) were found navigating the output of the Roary analysis.

## 4 Discussion

### 4.1 Florfenicol: Emergence of Resistance to RTFS'S Drug of Choice

Florfenicol (FLO), a fluorinated derivative of the naturally occurring chloramphenicol, is a broad‐range bacteriostatic synthetic drug and a potent ribosomal inhibitor solely used in veterinary medicine (Schwarz et al. 2004; Kohanski et al. 2010). Intrinsic resistance has not been detected. However, bacteria have evolved various mechanisms to circumvent its inhibiting effect such as efflux systems (Schwarz et al. 2004). In 2019, florfenicol was placed in the AMEG (Antimicrobial Advice *ad hoc* Expert Group) category C (‘Caution’) (Committee for Medicinal Products for Veterinary use (CVMP) and Committee for Medicinal Products for Human Use (CHMP) 2019). In DANMAP2023, an increased use of florfenicol has been reported in cattle < 1 year with a direct correlation to increased number of chloramphenicol‐resistant *E. coli* indicator bacteria (Andersen and Sandberg 2023). In Chile, the second largest producer of farmed salmon and with the highest rates of antimicrobial use per ton of harvested fish, florfenicol is most frequently used for the treatment of *Piscirickettsia salmonis* (Miranda et al. 2018).

In this study, we report for the first time the presence of a chloramphenicol/florfenicol efflux MFS transporter *floR* gene with high similarity to previously identified *floR* (in other bacteria) but of a longer sequence (413 aa instead of 404 aa) in two *Flavobacterium psychrophilum* FLO‐resistant isolates from Denmark (disc diffusion diameter of 7–8 mm and MIC > 16 μg mL^−1^). In addition, one of these isolates was associated with a case of ineffective florfenicol treatment in the field. Previous research focusing on AMR in *F. psychrophilum* and other closely related bacteria had found contrasting results between phenotypic and genotypic data, i.e., the *floR* gene (total length or partial sequence) was detected in florfenicol susceptible isolates (e.g., Saticioglu et al. (2019) and Verner‐Jeffreys et al. (2017)). In another study, two *F. psychrophilum* isolates from Chilean salmon farms were found with a reduced susceptibility to FLO (MIC of 16 and 32 μg mL^−1^) but without detecting any *floR* gene (Miranda et al. 2016). Other studies have observed a reduced susceptibility towards florfenicol (MIC > 2 μg mL^−1^) with a diverse range of MIC values in different countries, e.g., China (MIC range = 2–8 μg mL^−1^; CLSI VET04‐A2 guidelines 2014) (Li et al. 2021), Canada (MIC range = 2–16 μg mL^−1^; CLSI guidelines 2006) (Hesami et al. 2010), Chile (MIC range = 4–32 μg mL^−1^; CLSI guidelines 2006) (Henríquez‐Núñez et al. 2012), Czech Republic (MIC range = 2–8 μg mL^−1^; CLSI VET04‐A2 guidelines 2014) (Vaibarova et al. 2024). However, MIC data comparison between studies from different countries before the standardization/harmonization of the MIC determination protocol and its interpretative criteria for this bacterium by CLSI VET04‐A2 guidelines in 2014 is difficult (Smith et al. 2016). Nevertheless, no correlation between these phenotypes and the gene content was established.

In the current study, a positive correlation between the resistant phenotype and the presence of the *floR* gene was identified. A blastp search of the 413 aa sequence identified four proteins with high similarity and same length belonging to *Riemerella anatipestifer* isolates, Gram negative bacteria belonging to the family of Flavobacteriaceae and poultry pathogen (Chaves Hernández 2014), and *Chryseobacterium muglaense*, bacterium isolated from a fish farm in Turkey in 2015 (https://www.ncbi.nlm.nih.gov/protein/MBD3904429.1). This finding together with the observation of the different GC content of this gene located on a large genomic island also harbouring other genes related to antimicrobials and toxic compounds resistance might indicate its recent acquisition by horizontal gene transfer (HGT) and its possible further dissemination to other bacteria of the same species and/or other genera when the right conditions are encountered (e.g., selective pressure caused by antimicrobial use and close contact donor‐recipient bacteria). According to the literature (Schwarz et al. 2004), it is not uncommon to find *floR* together with other resistance genes within large mobile GIs able to chromosomally integrate and to remain stable. Examples are the SXT element in *Vibrio cholera* and the Salmonella Genomic Island 1 (SGI1) in *Salmonella enterica* , integrative conjugative elements of large dimensions carrying *floR* and other resistance determinants (Schwarz et al. 2004). In further studies, the activity of the *floR* gene should be experimentally confirmed by e.g., gene deletion technique (Barbier et al. 2020) and further characterization of MIC values (above 16 μg mL^−1^) should be included.

One of the isolates included in this study, which was related to a case of ineffective florfenicol treatment, showed reduced susceptibility to FLO in AST without harbouring any *floR* gene (values of disc diffusion and MIC determination were in vicinity of established resistance/NWT thresholds). As this finding resembles results of previous studies (discussed above), the presence of other undescribed mechanisms causing this reduced susceptibility is likely. Examples could be the presence of multi‐drug efflux pumps, the activity of certain enzymes inactivating the antimicrobial compound (e.g., O‐phosphorylation and hydrolytic degradation), the modification of the outer membrane e.g., by absence/loss of proteins, such as porins, that decreases membrane permeability, and finally mutations in ribosomal protein gene clusters (rare) (Schwarz et al. 2004). There could also be novel genes that are not currently described in the databases used.

### 4.2 Epidemiological Background, Serotype and Virulence: *floR* ‐Harbouring Isolates Belong to CC‐ST90, Previously Undetected in the Nordics, and Cause Disease in Experimental Infections

The evolution of the epidemic *F. psychrophilum* population (expansion of genetically closely related isolates) is driven mainly by recombination (Siekoula‐Nguedia et al. 2012; Nilsen et al. 2014) but also by mutation (Calvez et al. 2021), and the CC‐ST10 is the predominant clonal complex associated with disease in rainbow trout worldwide (Duchaud et al. 2018). In the Nordic countries (Sweden, Denmark, Finland and Norway), a study including 560 *F. psychrophilum* isolates, mostly from disease outbreaks, collected from 1983 to 2012 found the putatively virulent CC‐ST10 to be the most prevalent clonal complex (most prevalent STs in Denmark: ST2, ST10, ST79 and ST92), strengthening the hypothesis of its dissemination by commercial movement of fish or their products (Nilsen et al. 2014). In the current study, we identified three isolates from the same farm belonging to CC‐ST90 (ST: 90, 210; two harbouring the newly detected *floR* gene), previously not detected in the Nordic countries (Nilsen et al. 2014). Based on the PubMLST isolates database (Jolley et al. 2018) (October 2025), isolates belonging to this CC have been, for example, found in Chile (ST68; 7 from *O. mykiss* , 2 from *Salmo salar* and one unknown; kidney and gill samples), Switzerland (ST1, ST90 and ST210; 9 from *O. mykiss* and 1 from *S. trutta lacustris*; spleen samples), France (ST1, ST90 and ST108; 13 from *O. mykiss* ; internal and external organs), and Russia (ST329; one isolate from *O. mykiss* ; kidney sample) [publications about these isolates: (Siekoula‐Nguedia et al. 2012; Strepparava et al. 2013; Avendaño‐Herrera et al. 2014; Duchaud et al. 2018; Sundell et al. 2019)]. Isolates belonging to CC‐ST90 have been also found in Italy (2011, spleen of rainbow trout, ST90) (Duchaud et al. 2018) and recently in the Czech Republic, mostly belonging to ST329 (Vaibarova et al. 2024).

Interestingly, the two isolates collected from the same case (kidney of two diseased fish, serotype Th) were characterized by two different ST types (ST: 90, 210, both belonging to CC‐ST90) and opposite AMR features (OXY and FLO resistance) indicating the presence of more than one ST involved in this diagnostic case. This was also observed previously by Nilsen et al. (2014), where coinfection of different ST types on the same rainbow trout was detected and advice to examine more than one colony from infected fish was given. Co‐carriage of up to five STs and three serotypes was also detected in spleen and gills samples from an apparently healthy fish by Calvez et al. (2021) (avirulent or fish were latent carriers). As ST90 and ST210 have been previously identified in France, Italy (ST90) and Switzerland (ST90 and ST210), commercial movement of, for example, eyed eggs between European countries could be a likely source of these newly identified STs in Denmark. Poor disinfection procedures of eyed eggs, improper cleaning procedures of e.g., RAS systems between fish batches or introduction of bacteria via people or equipment during grading or daily procedures could be key players in the spreading of this bacterium between farms (Madsen et al. 2005; Madsen and Dalsgaard 2008). Also, broodfish are latent carriers of this pathogen and, without presenting any signs of disease, they can carry different ribotypes and serotypes (Madsen et al. 2005).

As discussed by Saticioglu et al. (2024), a complete understanding of the genomic determinants underlying varying levels of in vivo virulence as well as the development of in vitro assays capable of distinguishing among these degrees has yet to be achieved (Sundell et al. 2019; Castillo et al. 2021). Exceptions are those mutations/phenotypic anomalies in the Type‐9 Secretion System (T9SS) associated with a complete loss of virulence (Barbier et al. 2020; Jørgensen et al. 2022). in vivo characterization of median lethal dose (LD_50_) based on experimental models such as the one used by Sundell et al. (2019) allows a preliminary in vivo virulence assessment of a large panel of isolates while reducing the number of fish used. Therefore, we selected this infection model. Nevertheless, virulence should be further assessed with a more comprehensive in vivo set up in dubious cases. In this study, the animal experiments confirmed that all tested isolates caused disease in rainbow trout fry with LD_50_ values below 10^5^ CFU. Among the isolates belonging to CC‐ST90 and harbouring the *floR* gene, *F. psychrophilum* 21‐9705‐2 (serotype 2, ST210) was tested in such experiment and it showed an LD_50_ equal to 4.4 × 10^3^ CFU. For three isolates belonging to CC‐ST10, the selected range of bacterial doses (10^2^–10^7^ CFU fish^−1^) was insufficient for these estimations as they were characterized by LD_50_ values off scale, i.e., below the lower limit of 10^2^ CFU [20‐8139‐2B: serotype 1, ST2; 20‐1298‐14A: serotype 2, ST92; 21‐9697‐3A1: serotype 2, ST443]. At first glance the overall virulence of the current panel of isolates appears elevated in comparison with previous studies (Sundell et al. 2019). On the other hand, the LD_50_ values of Danish reference strains obtained in the current study were also lower compared to previous work (160401‐1/5N: 7.2 × 10^2^ CFU vs. 9.8 × 10^4^ CFU; 950106‐1/1: 3.8 × 10^5^ CFU vs. 2.6 × 10^6^ CFU) (Sundell et al. 2019). Variations in rainbow trout intra‐species diversity as well as fish size (5 g) and injection procedure (intramuscular injection) are the likely causes of this difference (Wiens et al. 2018; Lee et al. 2023). One pitfall of the model is the possibility of secondary infections as happened for two isolates included in the study (20‐1298‐14A and 20‐8139‐2B). Given the preliminary nature of these results, virulence should be further evaluated with a new in vivo experimental trial with a different design, i.e., more comprehensive range of bacterial doses where fish injected with each specific dose are kept separately in their own aquarium.

### 4.3 Considerations About Possible Antimicrobial Alternatives to Florfenicol: Oxytetracycline and Amoxicillin

In Danish RTFS cases where e.g., florfenicol is not a treatment option due to resistance, oxytetracycline (OXY) and amoxicillin (AMO) can occasionally be used: a veterinarian receives a dispensation after proper AST (Bruun et al. 2000; Dalsgaard 2014). Both antimicrobials belong to the AMEG category D (‘Prudence’) (Committee for Medicinal Products for Veterinary use (CVMP) and Committee for Medicinal Products for Human Use (CHMP) 2019).

OXY is a broad‐spectrum bacteriostatic agent targeting the 30S subunit of the bacterial ribosomes. OXY has been the drug of choice for many bacterial diseases in fish but because of its low absorption by the fish, the standard dose for treatment is higher compared to drug doses used in other animals. Also, resistance to this antimicrobial has risen dramatically since its introduction (Lunestad and Samuelsen 2008). In Denmark, OXY has been used for the treatment of RTFS for a short period when *F. psychrophilum* was initially isolated (1985), since its efficacy dropped in the early 90s (Bruun et al. 2000; Dalsgaard 2014). In the studied bacterial isolates, we saw OXY reduced susceptibility in AST (disc diffusion) in three isolates belonging to ST92 (CC‐ST10). However, we could not confirm these results on the broth microdilution AST because of QC MIC values being outside the recommended ranges. In addition, it was not possible to show a clear correlation with the gene content. For example, two of the isolates with reduced susceptibility to OXY harboured a putative Tet‐res gene in two exact copies, which was also found in one copy in the Danish reference strains, one susceptible and one with reduced susceptibility to OXY. Carried on plasmids or transposons, MFS efflux pumps are the main driver of tetracycline resistance, together with ribosomal protection proteins (Pearson et al. 2025). Further investigation of the putative Tet resistance genes should be performed in the future.

AMO is a semisynthetic β‐lactam antimicrobial with bacteriolytic activity and moderate‐spectrum (large range of Gram‐positive bacteria, limited in the case of Gram‐negative) (Salvo et al. 2009). For a short period of time, it was the drug of choice for RTFS in Denmark. However, the bacteria became quickly resistant (Bruun et al. 2000). In the current study, none of the tested isolates presented resistance to AMO or other β‐lactams in the phenotypic tests. The presence of a putative metal‐dependent hydrolase (Castillo et al. 2021) did not seem to interfere with AMO or other lactams in vitro susceptibility.

Finally, multidrug efflux pumps were identified in the tested isolates, i.e., an RND (resistance nodulation division family; all isolates) and an MFS (major facilitator superfamily; in 4 isolates) multidrug efflux transporters, which could be associated with resistance to a broad range of antimicrobials. Indeed, these systems can expel the antimicrobial or be involved in the acquisition of additional resistant mechanisms that decrease the antimicrobial concentration in the cell (Sun et al. 2014). Further studies are needed to shed light on the activity of these efflux pumps.

### 4.4 Resistance to Licensed Drugs Used for Other Bacterial Infections in Aquaculture: Oxolinic Acid

Oxolinic acid (OXO), broad‐spectrum antimicrobial with bactericidal activity against Gram‐negative bacteria, is one of the most used quinolones in aquaculture. By passive diffusion, OXO reaches the bacteria intracellularly and inhibits the DNA gyrase, core enzyme for DNA synthesis (Lunestad and Samuelsen 2008; Hooper and Jacoby 2016). In Denmark, OXO has been used as a licensed drug for bacterial infections in aquaculture, freshwater and marine farms since 1986 but not specifically for RTFS (Bruun et al. 2000). In Europe, OXO and other quinolones, e.g., enrofloxacin, belong to the AMEG category B (‘Restrict’) (Committee for Medicinal Products for Veterinary use (CVMP) and Committee for Medicinal Products for Human Use (CHMP) 2019).

Widespread among Gram‐positive and negative bacteria, one of the mechanisms of quinolone resistance (QR) is the alteration of the quinolone target enzymes: DNA gyrase (*gyrA* and *gyrB*) and DNA Topoisomerase IV (*parC* and *parE*) (Hooper and Jacoby 2016). The acquisition of mutations in the *gyrA* gene is usually responsible for QR to 1st generation quinolones (e.g., oxolinic acid), while a higher level of resistance is associated with mutations occurring simultaneously in *gyrA* and *gyrB* or *parC* (Shah et al. 2012). In the current study, the *gyrA* aa sequences of *F. psychrophilum* strains resistant to OXO were characterized by Thr82→Val/Ala/Arg aa substitution, corresponding to aa pos. 83 in *Escherichia coli* numbering, which falls in the Quinolone Resistance Determining Region of *gyrA* (QRDR; aa pos 67–106, *E. coli* numbering) (Hooper and Jacoby 2016). Similar results have been previously described in *F. psychrophilum* isolates in e.g., Norway (Shah et al. 2012), Japan (Izumi and Aranishi 2004), Sweden (Söderlund et al. 2018) and Turkey (Saticioglu et al. 2019). The fully susceptible/WT towards OXO isolate 22‐344‐3B presented missense mutations in *gyrA* outside the QRDR (Arg481→Cys). Interestingly, the isolate 21‐9705‐2 characterized by AST values on the thresholds for resistance towards OXO presented missense mutations in the genes *gyrA* (Arg481→Cys) and *parC* (Asp340→Asn), both outside the QRDR of the respective genes (QRDR *parC*: aa pos. 63–102, *E. coli* numbering) (Hooper and Jacoby 2016). The reduced susceptibility to OXO of this isolate could be related to the presence of other not identified mechanisms e.g., mutations in regulatory proteins controlling the expression of porins causing an increased efflux of the drug out of the cell (Hooper and Jacoby 2016).

All the analysed *F. psychrophilum* strains were characterized as NWT towards the fluoroquinolone enrofloxacin (ENR). In Denmark, this antimicrobial is registered for veterinary therapeutic use in animals, and, as the other fluoroquinolones, its use has been restricted through the legislation implemented in 2002. Currently, fluoroquinolones can only be used as the last‐defence antimicrobial in food‐producing animals after appropriate AST in an accredited lab (Andersen and Sandberg 2023). In Denmark, no fluoroquinolones have been used for the treatment of fish diseases. However, worldwide, six of the major 15 aquaculture‐producing countries use this antimicrobial for bacterial diseases outbreaks in aquaculture with different regulations depending on the country (Lulijwa et al. 2020).

### 4.5 Resistance to Licensed Drugs Used for Other Bacterial Infections in Aquaculture: Potentiated Sulphonamides

Potentiated sulphonamides, i.e., combined with a 2,4 diaminopyrimidine as e.g., trimethoprim, are synthetic DNA synthesis inhibitor drugs primarily targeting and inhibiting the tetrahydrofolic acid synthesis in both Gram negative and positive aerobic bacteria (Lunestad and Samuelsen 2008; Kohanski et al. 2010). In aquaculture, the combinations sulphadimethoxine with ormethoprim (5:1) [trade name: Romet] and sulfadiazine with trimethoprim (5:1) [trade name: Tribrissen] have been used for the treatment of enteric redmouth disease, furunculosis, vibriosis and enteric septicaemia (Lunestad and Samuelsen 2008) but not for RTFS. Sulfadiazine with trimethoprim belong to the AMEG category D (‘Prudence’) (Committee for Medicinal Products for Veterinary use (CVMP) and Committee for Medicinal Products for Human Use (CHMP) 2019).

Contrasting results and difficulties in AST data interpretation have been reported in relation to potentiated sulphonamides resistance and *Flavobacteriun psychrophilum* (Smith et al. 2016; Ngo et al. 2017; Saticioglu et al. 2019). Resistance to potentiated sulphonamides was initially thought to be an inherent character of this bacterium or a very widespread acquired feature (Bruun et al. 2000; Dalsgaard and Madsen 2000; Schmidt et al. 2000), as only a few isolates were found to be susceptible (Bruun et al. 2000). However, the same Danish isolates from Bruun et al. (2000) were categorized as WT towards TRS in a subsequent analysis (Smith et al. 2016). In Saticioglu et al. (2019), authors could not establish a link between the detection of the *sul*2 gene (PCR based detection, resistance to sulfonamides) and MIC values. In this study, the AST data obtained showed widespread resistance towards TRS except for three of the recent bacteria that were categorized as susceptible by disc‐diffusion assay. Obtained MIC values did not show a matching pattern with disc diffusion data. No ARGs in relation to TRS resistance were detected by submitting the genomes to either ResFinder or the CARD's RGI Finder. Also, no unique gene was found in TRS resistant isolates during pangenome analysis.

In Saticioglu et al. (2019), researchers hypothesized the presence of mutations in the chromosomal DHPS gene *folP* (dihydropteroate synthetase, enzyme inhibited by sulfonamides) as observed for other bacteria (Vedantam et al. 1998; Enne et al. 2002). In our isolates, there was no clear correlation between changes in the gene sequences of the isolates and their phenotypic profiles in relation to TRS. A similar scenario was observed when aligning the sequences of the DHFR genes (dihydrofolate reductase, enzyme inhibited by trimethoprim) (Sánchez‐Osuna et al. 2020).

### 4.6 Methodological Considerations/Limitations

For an appropriate characterization of susceptibility/resistance and to support a sensible comparison among studies, antimicrobial susceptibility testing (AST) should be performed following standardized guidelines. In this study, disc diffusion AST (1:7 diluted MH + 5% FBS) with historically comparable results (Bruun et al. 2000; Dalsgaard 2014; Miranda et al. 2016) and CLSI guidelines (CLSI 2020a) were followed. Broth‐micro dilution AST (CLSI 2020a) was added to support the disc diffusion results (Miranda et al. 2016; Smith et al. 2018), but a deviation from the CLSI suggested protocol was in this case needed: The recommended diluted CAMHB did not support the growth of the selected *F. psychrophilum* isolates in the custom‐made Sensititre AQGNECV dried panels in our tests while growth was instead observed in the QC strains and their MIC values were within the correct ranges.

Nevertheless, the results of the two methods aligned well overall. The effect of the medium change was observed for TRS and OXY, where the MIC values obtained for one (TRS) or both (OXY) of the QC strains were outside the recommended ranges. Indeed, Muller Hinton Broth (MHB) is recommended since, among other reasons, it is low in inhibitors that can alter the outcome in case of sulfonamide/trimethoprim and tetracycline (CLSI 2020a). When both QC strains were outside the recommended ranges (OXY), MIC values for that antimicrobial were not considered.

No CLSI‐official clinical breakpoints exist for *F. psychrophilum* (CLSI 2020a). In this study, we relied on historically established interpretative criteria for disc diffusion AST (Dalsgaard 2014), which were then well supported by the categorization in WT/NWT based on CLSI ECVs despite the inoculum medium modification. However, a larger panel of isolates covering a larger spatio‐temporal frame should be tested in the future to evaluate if any update should be done to the historically established interpretation criteria for disc diffusion AST. Further evaluations in regard to the growth issues observed in CAMHB should also be made to better adhere to broth micro‐dilution CLSI guidelines.

## 5 Conclusion and Future Perspectives

In this study, we evaluated the antimicrobial susceptibility profile of a small panel of *Flavobacterium psychrophilum* isolates collected in Danish rainbow trout farms in the period 2019–2022 and observed changes in the susceptibility to drugs currently in use in Danish aquaculture. Importantly, we detected reduced susceptibility to florfenicol, drug of choice for RFTS/BCWD treatment in Denmark since 1996, in a diagnostic case associated with clinical disease and ineffective antibiotic treatment in one freshwater farm. The in vitro pattern was again observed in subsequent clinical outbreaks in the same facility, where the veterinarian asked for AST to support a correct use of antimicrobials. The genome analysis revealed the presence of a *floR* gene putatively conferring this property and these isolates belonged to a clonal complex not previously observed in Nordic studies (CC‐ST90). To the authors knowledge, this is the first report describing phenotypic florfenicol resistance in *floR*‐harbouring *Flavobacterium psychrophilum* . However, the activity of the *floR* gene must be further characterized with in vitro lab experiments, and the FLO resistance patterns further confirmed with experimental in vivo studies where the efficacy of the antimicrobial treatment is tested in experimentally challenged fish with *F. psychrophilum* isolates with different in vitro antibiotic susceptibility. This type of experiment would support the clinical significance of the NWT in vitro phenotypes i.e., the clinical relevance of e.g., the *floR*‐harbouring isolates showing in vitro reduced florfenicol susceptibility (Miranda et al. 2016). An example is the work done by Bruun et al. (2003), where the validity of predicting the treatment efficacy of OXY from in vitro AST was demonstrated.

Due to the absence of monitoring/surveillance programs concerning AMR in fish pathogens in Denmark, an overall understanding of the florfenicol resistance/reduced susceptibility distribution in the field is missing. In our laboratory, diagnostic samples do not represent all RTFS outbreaks in the field, but they instead relate to cases where the veterinarian has requested further analysis such as in vitro AST to support a prudent use of antimicrobials. Diagnostic cases related to ineffective florfenicol treatment on a diseased batch of fish have been seen only in one farm over the past 5 years. In the same farm, RTFS cases before 2020 were efficiently treated with florfenicol.

Despite this investigation being done on few isolates, the findings raise the question of whether the observed changes might be indicative of wider trends across farms or regions and/or be related to the commercial movement of fish at different life stages (e.g., eyed eggs) from/to other countries. The increasing number of RAS farms and the intensive production, together with the commercial movement of fish, could contribute to the dissemination of this resistance pattern. Furthermore, the location of the *floR* gene, putatively conferring the resistance, within a large genomic island represents a prerequisite for its dissemination and if combined with an appropriate selective pressure (use of florfenicol or other antimicrobials) and a close contact donor‐recipient bacteria, a fast and efficient spreading of *floR* to other bacteria could happen (same species or other species or genera) (Schwarz et al. 2004). Thus, the characterization of a larger panel of isolates covering a wider spatio‐temporal frame would be beneficial to better understand the distribution of this newly observed pattern.

## Author Contributions

**Valentina L. Donati:** conceptualization, investigation, writing – original draft, methodology, validation, visualization, writing – review and editing, software, formal analysis, data curation. **Lone Madsen:** conceptualization, investigation, methodology, writing – review and editing, resources, funding acquisition, project administration.

## Funding

‘Sundt Dambrug: Reduced use and release of environmentally damaging antibiotics from fish farms’ project with grant no. 33111‐I‐21‐082 is co‐funded by the European Union through the European Maritime and Fisheries Fund (EMFF) and the ‘Sund Akvakultur: Aquaculture without antibiotics' project with grant no. GOA‐UDV‐23‐0017 is co‐funded by the European Union through the European Maritime, Fisheries and Aquaculture Fund (EMFAF).

## Conflicts of Interest

The authors declare no conflicts of interest.

## Supporting information


**Table S1:** Sequencing statistics and genome QC of the newly sequenced *F. psychrophilum* isolates. Data (Mbp) and No. of reads: sequencing data yield in total base pairs and number of reads, respectively. Read N50: half of the data is contained between reads of length N50 or larger. Q‐score: median Phred scaled read quality score. Completion (%): estimate genome completeness based on presence/absence of essential lineage‐specific marker genes. Contamination (%): estimated contamination based on the presence of multiple single‐copy marker genes. Strain heterogeneity (%): estimated strain heterogeneity based on the number of multi‐copy marker pairs which exceed a specific amino acid identity threshold. GTDB taxonomy: it refers to the GTDB (Genome Taxonomy Database) classification at the highest resolution assigned to a specific genome. Phred‐scaled assembly quality value (QV) is calculated as −10log_10_(error rate) where the error rate equals the ratio between the number of retained residual variants and the assembly size.
**Table S2:** Basic genome assembly statistics and accession numbers. The accession numbers of previously sequenced isolates used as references for the analysis are also included. Sequences are deposited under the NCBI bioproject PRJNA1165645. Unusual large plasmids are indicated in italics as they could be an artefact of ONT seq. and, their sizes were not confirmed experimentally. *For the isolate 950106‐1/1, its genome has been previously sequenced with both Illumina (CP008902.1) and PacBio (CP059135.1) technologies. The Illumina sequenced genome (CP008902.1) was used in this context to correct uncertainties related to possible sequencing errors in PacBio. The isolate FPS‐R7 was included as representative isolate carrying previously identified genes related to antibiotic/toxic compounds resistance not commonly found in reference strains (Castillo et al. 2021).
**Table S3:** Blastp search of MFS transporter protein sequence. The first 10 best matches are presented. The search was done in March 2023.
**Figure S1:** Presence/absence of genes involved in resistance to antibiotics and toxic compounds and types of mutations. Mutations compared to the reference strain *F. psychrophilum* FPG3 (or FPS‐R7 when not present in FPG3) are illustrated. For the gene identified by ResFinder/CARD‐RGI Finder, mutations compared to the databases sequences are illustrated. Loci numbers are indicated. **: In Fp 950106‐1/1, the *par*E gene presented a frameshift mutation (locus H0H25_RS00985), which was considered a sequencing error since absent when sequenced with Illumina (locus IB65_03145 in CP008902.1); A silent mutation was found by both seq. technologies. A similar situation was observed when mapping FPG3_06480 in 950106‐1/1 (PacBio: locus H0H25_RS00105; Illumina: IB65_04035).
**Figure S2:** Visualization of IslandViewer 4 results for *floR*‐harbouring isolates: (A) 21‐9705‐2 and (B) 22‐344‐3B. For both genomes, the largest genomic island (GI) predicted by both IslandPath‐DIMOB (in blue) and SIGH‐HMM (in orange) is highlighted in red in the small table to the left, in light grey on the circular genome representations and then zoomed in the lower genome panel. The positioning of the *floR* gene is highlighted in the lower genome panels as well as in the Tables to the right (in grey).
**Figure S3:** Multiple alignment of DHPS or *folP* (A) and DHFR (B) translated genes. *F. psychrophilum* FPG3 is used as reference for the alignments. Multiple alignment was performed with Geneious Prime. All disagreements are highlighted with the corresponding aa mismatch. In case of similarity, dots are used. The stop codon is represented by *. To the left, the in vitro susceptibility profile (disc diffusion AST) of each isolate towards SXT is presented: susceptible/WT phenotype (grey), resistant/NWT phenotype (red) and intermediate resistance (yellow). Intermediate resistance represents cases where obtained values were in the vicinity of resistance/NWT thresholds. DHPS or *folP*, dihydropteroate synthase; DHFR, dihydrofolate reductase.
**Figure S4:** Survival curves of fish infected with *F. psychrophilum* isolates for LD_50_ estimation [Kaplan–Meier survival analysis]. Rainbow trout fry ( *Oncorhynchus mykiss* ; 1–2 g) were selected for the experiments. For each of the seven isolates, two aquaria were set up (duplicates, 14 aquaria in total) and, in each aquarium, fish infected with six different doses with a separation factor of 10 (from 10^7^ to 10^2^ CFU fish^−1^) of the same isolate were placed together with negative control fish (sterile TYES‐B) (6 doses + negative control, 6 fish/dose, 42 fish/aquarium, 2 aquaria/isolate). For each bacterial dose, a specific type of fin clipping was performed to allow fish recognition during the trial. Negative control fish were not fin clipped. The well‐characterized Danish *F. psychrophilum* isolates 950106‐1/1 and 160401‐1/5N were included as quality controls. Intraperitoneal injection was the chosen infection method. Survival data from singular replicate aquaria were merged. Moribund/dead fish were all positive to *F. psychrophilum* . LD_50_ was estimated with the Reed‐Muench method (Reed and Muench 1938). The method requires observation of cumulative mortality above and below 50% among the infected groups.

**File S1:** Output of RGI Finder.

## Data Availability

The original contributions presented are included in the article. Assembled genomes can be found under the NCBI Bioproject PRJNA116564. Further inquiries can be directed at the corresponding author.
