# Supplementary material for: Phenotypic Florfenicol Resistance in floR‐Harbouring Flavobacterium psychrophilum Isolates (CC‐ST90): A Case Study From Danish Freshwater Rainbow Trout Aquaculture
Source: J Fish Dis. 2026 Jul 23;49(12):e70255. doi: 10.1111/jfd.70255 (PMC13749313; doi:10.1111/jfd.70255)
Supplement: Supplementary file 1 [file JFD-49-e70255-s002.docx]

**Supplementary material**

**Phenotypic florfenicol resistance in *floR*-harboring *Flavobacterium psychrophilum* isolates (CC-ST90): a case study from Danish freshwater rainbow trout aquaculture.**

Valentina L. Donati^1*^ and Lone Madsen^1^

Overview – Supplementary Tables

**Supplementary Table 1. Sequencing statistics and genome QC of the newly sequenced *F. psychrophilum* isolates.**

**Supplementary Table 2. Basic genome assembly statistics and accession numbers.**

**Supplementary Table 3. Blastp search of MFS transporter protein sequence.**

Overview – Supplementary Figures

**Supplementary Figure 1. Presence/absence of genes involved in resistance to antibiotics and toxic compounds and types of mutations.**

**Supplementary Figure 2.** **Visualization of IslandViewer 4 results for *floR*-harboring isolates: (A) 21-9705-2 and (B) 22-344-3B.**

**Supplementary Figure 3. Multiple alignment of DHPS or *folP* (A) and DHFR (B) translated genes.**

**Supplementary Figure 4. Survival curves of fish infected with *F. psychrophilum* isolates for LD_50_ estimation [Kaplan–Meier survival analysis].**

**Supplementary Table 1. Sequencing statistics and genome QC of the newly sequenced *F. psychrophilum* isolates.** Data (Mbp) and No. of reads: sequencing data yield in total base pairs and number of reads, respectively. Read N50: half of the data is contained between reads of length N50 or larger. Q-score: median Phred scaled read quality score. Completion (%): estimate genome completeness based on presence/absence of essential lineage-specific marker genes. Contamination (%): estimated contamination based on the presence of multiple single-copy marker genes. Strain heterogeneity (%): estimated strain heterogeneity based on the number of multi-copy marker pairs which exceed a specific amino acid identity threshold. GTDB taxonomy: it refers to the GTDB (Genome Taxonomy Database) classification at the highest resolution assigned to a specific genome. Phred-scaled assembly quality value (QV) is calculated as −10log10(error rate) where the error rate equals the ratio between the number of retained residual variants and the assembly size.

|  | **Seq statistics: Raw** | | | **Seq statistics: Trimmed** | | | | ***De novo* assembly: Genome QC** | | | | |
| --- | --- | --- | --- | --- | --- | --- | --- | --- | --- | --- | --- | --- |
| **Isolate** | **Data (Mbp)** | **Read N50 (bp)** | **q-score** | **No. of reads** | **Data (Mbp)** | **Read N50 (bp)** | **q-score** | **Completion (%)** | **Contamination (%)** | **Strain heterogeneity (%)** | **GTDB taxonomy** | **Phred-scaled assembly QV** |
| 22-344-2B | 432 | 9321 | 18.0 | 63187 | 394 | 9362 | 19.1 | 98.92 | 0.71 | 0 | s_Flavobacterium psychrophilum | 57.49 |
| 22-344-3B | 584 | 7968 | 17.8 | 94952 | 531 | 7956 | 19.0 | 98.20 | 0.71 | 0 | s_Flavobacterium psychrophilum | 53.43 |
| 21-9705-2 | 550 | 8444 | 17.8 | 84515 | 499 | 8442 | 19.0 | 98.82 | 0.71 | 0 | s_Flavobacterium psychrophilum | 51.94 |
| 20-8139-2B | 1231 | 7381 | 18.0 | 211121 | 1117 | 7370 | 19.2 | 99.29 | 0.44 | 0 | s_Flavobacterium psychrophilum | 60+ |
| 21-9697-3A1 | 856 | 7734 | 18.0 | 141388 | 780 | 7737 | 19.3 | 99.12 | 0.44 | 0 | s_Flavobacterium psychrophilum | 57.49 |
| 20-1298-14A G | 562 | 7692 | 17.9 | 91947 | 511 | 7670 | 19.0 | 99.29 | 0.44 | 0 | s_Flavobacterium psychrophilum | 60+ |
| 19-12880-80A | 855 | 6434 | 18.0 | 158724 | 777 | 6418 | 19.3 | 99.29 | 0.44 | 0 | s_Flavobacterium psychrophilum | 64.61 |

**Supplementary Table 2. Basic genome assembly statistics and accession numbers.** The accession numbers of previously sequenced isolates used as references for the analysis are also included. Sequences are deposited under the NCBI bioproject PRJNA1165645. Unusual large plasmids are indicated in italics as they could be an artifact of ONT seq. and, their sizes were not confirmed experimentally. * For the isolate 950106-1/1, its genome has been previously sequenced with both Illumina (CP008902.1) and PacBio (CP059135.1) technologies. The Illumina sequenced genome (CP008902.1) was used in this context to correct uncertainties related to possible sequencing errors in PacBio. The isolate FPS-R7 was included as representative isolate carrying previously identified genes related to antibiotic/toxic compounds resistance not commonly found in reference strains (Castillo et al., 2021).

| **Isolate** | **Genome size (bp)** | **GC content (%)** | **Genes (total)** | **CDS (total)** | **Genes (coding) /CDS (with protein)** | **rRNAs (16S, 23S, 5S)** | **tRNA** | **Accession no. genome** | **Other elements (bp)** | **Accession no. other elements** | **Source or publication** |
| --- | --- | --- | --- | --- | --- | --- | --- | --- | --- | --- | --- |
| 22-344-2B | 2,805,016 | 32.7 | 2,508 | 2,436 | 2,363 | 6, 6, 6 | 50 | CP170531 | - | - | This study |
| 22-344-3B | 2,860,897 | 32.7 | 2,577 | 2,505 | 2,423 | 6, 6, 6 | 50 | CP170530 | - | - | This study |
| 21-9705-2 | 2,812,909 | 32.8 | 2,527 | 2,455 | 2,366 | 6, 6, 6 | 50 | CP170537 | - | - | This study |
| 20-8139-2B | 2,806,172 | 32.6 | 2,500 | 2,429 | 2,404 | 6, 6, 6 | 49 | CP170535 | *10,221* | CP170536 | This study |
| 21-9697-3A1 | 2,789,698 | 32.5 | 2,503 | 2,432 | 2,401 | 6, 6, 6 | 49 | CP170542 | *13,634* | CP170543 | This study |
| 20-1298-14A G | 2,925,042 | 32.5 | 2,616 | 2,545 | 2,519 | 6, 6, 6 | 49 | CP170541 | 3,349 | CP170540 | This study |
| 19-12880-80A | 2,878,538 | 32.5 | 2,570 | 2,499 | 2,467 | 6, 6, 6 | 49 | CP170534 | *10,047*  2,107 | CP170532  CP170533 | This study |
| 160401-1/5N | 2,827,614 | 32.5 | 2,505 | 2,434 | 2,405 | 6, 6, 6 | 49 | CP039120.2 | 2,943 | NZ_CP059092.1 | (1, 2) *(Download: 19-01-2023)* |
| 950106-1/1* | 2,839,750 | 32.5 | 2,507 | 2,436 | 2,367 | 6, 6, 6 | 49 | CP059135.1 + CP008902.1 | 3,360 | MZ027733.1 | (2, 3)  *(Download:*  *19-01-2023)* |
| NCIMB1947 [FPG3 (ATCC 49418)] | 2,715,909 | 32.5 | 2,397 | 2,332 | 2,305 | 6, 6, 6 | 49 | CP007207.1 | 2,602 | - | (4)  *(Download:*  *07-03-2023)* |
| FPS-R7 | 3,208,574 | 32.6 | 2,888 | 2,816 | 2,795 | 7, 6, 6 | 49 | NZ_CP059075 | - | - | (1, 2) *(Download: 19-01-2023)* |

References:

1.	Sundell K, Landor L, Nicolas P, Jørgensen J, Castillo D, Middelboe M, Dalsgaard I, Donati VL, Madsen L, Wiklund T. 2019. Phenotypic and genetic predictors of pathogenicity and virulence in Flavobacterium psychrophilum. Front Microbiol 10:1–14.

2.	Castillo D, Donati VL, Jørgensen J, Sundell K, Dalsgaard I, Madsen L, Wiklund T, Middelboe M. 2021. Comparative genomic analyses of flavobacterium psychrophilum isolates reveals new putative genetic determinants of virulence traits. Microorganisms 9.

3.	Castillo D, Christiansen RH, Dalsgaard I, Madsen L, Middelboe M. 2015. Bacteriophage resistance mechanisms in the fish pathogen Flavobacterium psychrophilum: Linking genomic mutations to changes in bacterial virulence factors. Applied and Environmental Microbiology 81:1157–1167.

4.	Wu AK, Kropinski AM, Lumsden JS, Dixon B, MacInnes JI. 2015. Complete genome sequence of the fish pathogen Flavobacterium psychrophilum ATCC 49418T. Standards in Genomic Sciences 10:3.

**Supplementary Table 3. Blastp search of MFS transporter protein sequence.** The first 10 best matches are presented. The search was done in March 2023.

| **Description** | **Scientific Name** | **Max Score** | **Total Score** | **Query Cover** | **E value** | **Per. ident** | **Acc. Len** | **Accession** | **Identical proteins in strains**  **[isolation info: country, year, source]** |
| --- | --- | --- | --- | --- | --- | --- | --- | --- | --- |
| MFS transporter | *Flavobacterium psychrophilum* | 779 | 779 | 100% | 0.0 | 100.00% | 395 | [WP_094179087.1](https://www.ncbi.nlm.nih.gov/protein/WP_094179087.1?report=genbank&log$=prottop&blast_rank=1&RID=3AHZ5EE1013) | FPS-R7 [Russia, 2017, *O. mykiss*];  LM-02-Fp [Chile, 2006, kidney of *O. mykiss*];  96233 [Czech Republic, 2014, spleen of *O. mykiss*] |
| MFS transporter | *Flavobacterium psychrophilum* | 778 | 778 | 100% | 0.0 | 99.75% | 395 | [WP_221924667.1](https://www.ncbi.nlm.nih.gov/protein/WP_221924667.1?report=genbank&log$=prottop&blast_rank=2&RID=3AHZ5EE1013) | CN46 [China, 2020, *Thymallus thymallus*] |
| MFS transporter | Flavobacteriales bacterium | 701 | 701 | 99% | 0.0 | 92.89% | 395 | [MBL7940720.1](https://www.ncbi.nlm.nih.gov/protein/MBL7940720.1?report=genbank&log$=prottop&blast_rank=3&RID=3AHZ5EE1013) | new-MAG316 [China, 2020, bioreactor sludge-metagenome] |
| MFS transporter | *Flavobacterium psychrophilum* | 700 | 700 | 99% | 0.0 | 92.89% | 395 | [WP_227029159.1](https://www.ncbi.nlm.nih.gov/protein/WP_227029159.1?report=genbank&log$=prottop&blast_rank=4&RID=3AHZ5EE1013) | 2504s [Czech Republic, 2016, spleen of *O. mykiss*];  96239 [Czech Republic, 2016, kidney of *O. mykiss*];  97563, 97564, 97567, 97568, 97569, 97570, 97571, 97572, 97574, 97579, 97581, 97583, 97584, 97587, 97588 [Czech Republic, 2015, body cavity of *O. mykiss*];  jikry 4, jikry 5 [Czech Republic, 2018, eggs of *O. mykiss*]; 98244s, 98247s, 98253s, 98256s, 98752s [Czech Republic, 2015, spleen of *O. mykiss*];  9471s [Czech Republic, 2019, spleen of O*. mykiss*]; |
| MFS transporter | *Flavobacterium psychrophilum* | 691 | 691 | 99% | 0.0 | 85.53% | 395 | [WP_094163385.1](https://www.ncbi.nlm.nih.gov/protein/WP_094163385.1?report=genbank&log$=prottop&blast_rank=5&RID=3AHZ5EE1013) | KU 060626-4 [Japan, 2006, kidney of *Plecoglossus altivelis*] |
| MFS transporter | Bacteroidota bacterium | 668 | 668 | 99% | 0.0 | 88.58% | 395 | [MBS1775981.1](https://www.ncbi.nlm.nih.gov/protein/MBS1775981.1?report=genbank&log$=prottop&blast_rank=6&RID=3AHZ5EE1013) | Info not collected |
| MFS transporter | Bacteroidota bacterium | 665 | 665 | 99% | 0.0 | 88.07% | 395 | [MBS1640829.1](https://www.ncbi.nlm.nih.gov/protein/MBS1640829.1?report=genbank&log$=prottop&blast_rank=7&RID=3AHZ5EE1013) | Info not collected |
| putative tetracycline  resistant protein | uncultured bacterium | 627 | 627 | 99% | 0.0 | 77.66% | 396 | [ALG03693.1](https://www.ncbi.nlm.nih.gov/protein/ALG03693.1?report=genbank&log$=prottop&blast_rank=8&RID=3AHZ5EE1013) | Info not collected |
| MFS transporter | *Flavobacterium filum* | 625 | 625 | 98% | 0.0 | 82.73% | 395 | [WP_276974270.1](https://www.ncbi.nlm.nih.gov/protein/WP_276974270.1?report=genbank&log$=prottop&blast_rank=9&RID=3AHZ5EE1013) | Info not collected |
| MFS transporter | [Bacteroidia bacterium](https://www.ncbi.nlm.nih.gov/Taxonomy/Browser/wwwtax.cgi?id=2044936) | 625 | 625 | 99% | 0.0 | 78.93% | 394 | [HKC67596.1](https://www.ncbi.nlm.nih.gov/protein/HKC67596.1?report=genbank&log$=prottop&blast_rank=10&RID=EJH4143T016) | Info not collected |

**Supplementary Figure 1. Presence/absence of genes involved in resistance to antibiotics and toxic compounds and types of mutations**. Mutations compared to the reference strain *F. psychrophilum* FPG3 (or FPS-R7 when not present in FPG3) are illustrated. For the gene identified by ResFinder/CARD-RGI Finder, mutations compared to the databases sequences are illustrated. Loci numbers are indicated. **: In Fp 950106-1/1, the *par*E gene presented a frameshift mutation (locus H0H25_RS00985), which was considered a sequencing error since absent when sequenced with Illumina (locus IB65_03145 in CP008902.1); A silent mutation was found by both seq. technologies. A similar situation was observed when mapping FPG3_06480 in 950106-1/1 (PacBio: locus H0H25_RS00105; Illumina: IB65_04035).

**Supplementary Figure 2.** **Visualization of IslandViewer 4 results for *floR*-harboring isolates: (A) 21-9705-2 and (B) 22-344-3B.** For both genomes, the largest genomic island (GI) predicted by both IslandPath-DIMOB (in blue) and SIGH-HMM (in orange) is highlighted in red in the small table to the left, in light grey on the circular genome representations and then zoomed in the lower genome panel. The positioning of the *floR* gene is highlighted in the lower genome panels as well as in the Tables to the right (in grey).

**Supplementary Figure 3. Multiple alignment of DHPS or *folP* (A) and DHFR (B) translated genes.** *F. psychrophilum* FPG3 is used as reference for the alignments. Multiple alignment was performed with Geneious Prime. All disagreements are highlighted with the corresponding aa mismatch. In case of similarity, dots are used. The stop codon is represented by *. To the left, the *in vitro* susceptibility profile (disc diffusion AST) of each isolate toward SXT is presented: susceptible/WT phenotype (grey), resistant/NWT phenotype (red) and intermediate resistance (yellow). Intermediate resistance represents cases where obtained values were in the vicinity of resistance/NWT thresholds. DHPS or *folP*: dihydropteroate synthase. DHFR: dihydrofolate reductase.

**Supplementary Figure 4. Survival curves of fish infected with *F. psychrophilum* isolates for LD_50_ estimation [Kaplan–Meier survival analysis].** Rainbow trout fry (*Oncorhynchus mykiss;* 1-2 g) were selected for the experiments. For each of the seven isolates, two aquaria were set up (duplicates, 14 aquaria in total) and, in each aquarium, fish infected with six different doses with a separation factor of 10 (from 10^7^ to 10^2^ CFU fish^–1^) of the same isolate were placed together with negative control fish (sterile TYES-B) (6 doses + negative control, 6 fish/dose, 42 fish/aquarium, 2 aquaria/isolate). For each bacterial dose, a specific type of fin clipping was performed to allow fish recognition during the trial. Negative control fish were not fin clipped. The well-characterized Danish *F. psychrophilum* isolates 950106-1/1 and 160401-1/5N were included as quality controls. Intraperitoneal injection was the chosen infection method. Survival data from singular replicate aquaria were merged. Moribund/dead fish were all positive to *F. psychrophilum*. LD_50_ was estimated with the Reed-Muench method (Reed and Muench, 1938). The method requires observation of cumulative mortality above and below 50% among the infected groups.
